# PD-L1 checkpoint blockade delivered by retroviral replicating vector confers anti-tumor efficacy in murine tumor models

**DOI:** 10.18632/oncotarget.26785

**Published:** 2019-03-19

**Authors:** Leah A. Mitchell, Kader Yagiz, Andrew Hofacre, Sophie Viaud, Anthony W. Munday, Fernando Lopez Espinoza, Daniel Mendoza, Maria E. Rodriguez-Aguirre, Simon Bergqvist, Ali Haghighi, Marin V. Miner, William P. Accomando, Cynthia Burrascano, Dawn Gammon, Harry E. Gruber, Douglas J. Jolly, Amy H. Lin

**Affiliations:** ^1^ Tocagen Inc., San Diego, 92121, CA, USA; ^2^ Biofizik, Inc., San Diego, 92121, CA, USA

**Keywords:** immunotherapy, single chain variable fragment, retroviral replicating vector, PD-L1, PD-1

## Abstract

Immune checkpoint inhibitors (CPIs) are associated with a number of immune-related adverse events and low response rates. We provide preclinical evidence for use of a retroviral replicating vector (RRV) selective to cancer cells, to deliver CPI agents that may circumvent such issues and increase efficacy. An RRV, RRV-scFv-PDL1, encoding a secreted single chain variable fragment targeting PD-L1 can effectively compete with PD-1 for PD-L1 occupancy. Cell binding assays showed trans-binding activity on 100% of cells in culture when infection was limited to 5% RRV-scFv-PDL1 infected tumor cells. Further, the ability of scFv PD-L1 to rescue PD-1/PD-L1 mediated immune suppression was demonstrated in a co-culture system consisting of human-derived immune cells and further demonstrated in several syngeneic mouse models including an intracranial tumor model. These tumor models showed that tumors infected with RRV-scFv-PD-L1 conferred robust and durable immune-mediated anti-tumor activity comparable or superior to systemically administered anti-PD-1 or anti PD-L1 monoclonal antibodies. Importantly, the nominal level of scFv-PD-L1 detected in serum is ∼50–150 fold less than reported for systemically administered therapeutic antibodies targeting immune checkpoints. These results support the concept that RRV-scFv-PDL1 CPI strategy may provide an improved safety and efficacy profile compared to systemic monoclonal antibodies of currently approved therapies.

## INTRODUCTION

A retroviral replicating vector (RRV) platform based on an amphotropic gamma retrovirus that preferentially infects and replicates in tumor cells has been developed to address historical challenges with viral-based treatments for cancer. One example of the RRV approach is Toca 511, (vocimagene amiretrorepvec), an RRV encoding optimized cytosine deaminase derived from yeast) used in combination with an oral prodrug, Toca FC (extended-release 5-fluorocytosine) which has demonstrated durable, complete responses and a favorable safety profile in a Phase 1 trial [1] and is currently in a fully enrolled Phase 3 trial in patients with recurrent high grade glioma (NCT02414165). In addition to its tumor-selectivity, integration of RRV genome into the cancer cell genome allows sustained and localized therapeutic transgene expression in the tumor microenvironment and substantial bystander effects [2, 3]. Furthermore, unlike oncolytic viruses, RRV’s non-lytic replication process does not trigger immediate anti-viral immune responses, allowing for sustained viral replication and therapeutic transgene expression in the tumor microenvironment [4, 5].

Immune checkpoint inhibitors (CPI) such as therapeutic antibodies targeting cytotoxic T lymphocyte antigen 4 (CTLA-4), programmed death-1 (PD-1) and its ligand programmed death-1 ligand 1 (PD-L1) have represented a breakthrough for immunotherapies in treating cancer patients and have specifically made a significant impact in the fight against melanoma, lung, bladder head and neck cancer as well as in Hodgkin lymphoma [6]. However, there is still a substantial medical need that remains; the response rate ranges from 20–30% across tumor types with a number of immune-related adverse events associated with treatment to a high rate of treatment discontinuation (∼12–39% patients) [7–9]. In addition, among the approved CPIs, although PD-1 receptor occupancy has been demonstrated in circulating T cells following anti-PD-1 therapy [10, 11], it is unclear if complete saturation of PD-1 in circulating T cells serves as a surrogate biomarker for PD-1/L1 blockade in the tumor microenvironment (TME) [12, 13].

PD-1 is one of the major inhibitory receptors highly expressed on tumor infiltrating lymphocytes where upon its ligation with PD-L1 reduces cytokine production and T-cell proliferation resulting in immune escape from an active anti-tumor T-cell response in the TME [14]. In addition to the immune suppression mediated by PD-1/PD-L1 ligation, PD-L1 can also bind to CD80/B7.1 expressed on antigen presenting cells and block activation of T-cells through CD80/B7.1 binding to CD28 [15, 16]. PD-L1 expression is found in a broad range of tissues including T and B cells, dendritic cells, and macrophages [17]. PD-L1 upregulation is also found in tumor cells across many cancer types [18, 19]. Its expression is associated with MAPK or PI3K oncogenic pathways and is induced by IFN-γ thus representing an acquired mechanism of immune evasion [19, 20]. As PD-L1 is a proven immuno-oncology target for many cancer types and its expression is not limited to cancer cells in the TME, use of an RRV to selectively deliver a PD-1/L1 blockade in the TME may have desirable properties.

We have previously reported a new RRV configuration utilizing the “self-cleavage” 2A peptide (RRV-2A) as an alternative of the internal ribosomal entry sequence (IRES) described in Toca 511 [21, 22]. The RRV-2A configuration allows insertion of therapeutic transgene(s) up to approximately 1.2 kb and high efficiency of polyprotein separation. Also, RRV-2A tolerates single- and multiple-therapeutic transgene insertion without compromising the viral genome stability, infectivity and transgene expression [21]. In the current study, we explored a strategy using RRV-2A encoding a secreted single-chain variable fragment targeting PD-L1 (RRV-scFv-PDL1) to deliver an immune CPI that may reduce systemic immune toxicities and enhance efficacy through selective delivery to the TME. Although oncolytic viruses encoding immune CPIs have been reported, the efficacy in delaying tumor progression is limited [23]. Here, we demonstrate that RRV-scFv-PDL1 exhibits normal viral assembly and functions with a high level of genome stability. We also demonstrate that scFv PD-L1 effectively block PD-1/PD-L1 interaction leading to alleviation of PD-1/PD-L1 mediated immune suppression in a co-culture system *in vitro.* This activity was further supported by data from multiple syngeneic mouse models showing that tumors infected with RRV-scFv-PDL1 conferred robust and durable immune-mediated anti-tumor activity comparable or superior to systemically administered anti-PD1 and anti-PD-L1 monoclonal antibodies. Most importantly, our data show that only nominal level of scFv-PD-L1 was found in the circulation in mouse models where significant efficacy was observed. These results support the concept of an RRV-based platform for delivery of an immune CPI for an improved therapeutic window compared to systemic monoclonal antibodies currently approved for clinical use in many cancer types.

## RESULTS

### scFv PD-L1 encoded in the RRV-2A configuration is expressed and properly processed

**Figure 1:**
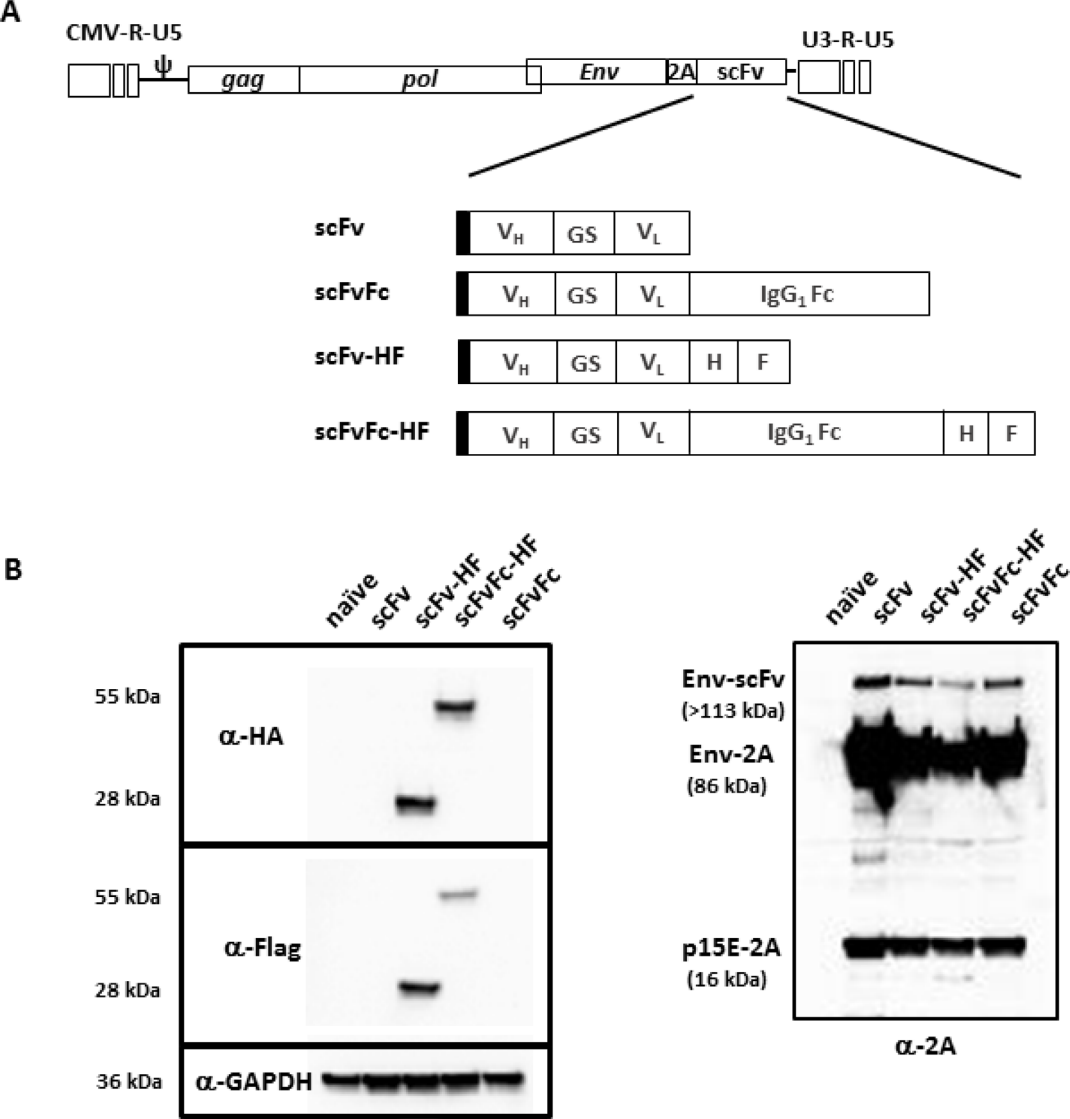
Schematic diagram of RRV-scFv-PDL1 plasmid DNAs (**A**) Two pairs of single-chain variable fragment (scFv) against PD-L1 were encoded in pAC3 RRV backbone. One pair consists of scFv with and without the Fc from human IgG1, designated as pAC3-scFv-PDL1 and pAC3-scFvFc-PDL1, respectively. Another pair consists of scFv-PDL1 and scFvFc-PDL1 with HA and Flag epitope incorporated at the C-terminus, designated as pAC3scFvHF-PDL1, pAC3-scFvFc-HF-PDL1. Filled rectangle indicates leader sequence derived from human IL-2. (**B**) Western blot analysis of viral envelope proteins produced transient transfection in 293T cells. Twenty micrograms of total protein lysates were loaded per well. Membranes were incubated (left panel) with anti-HA and anti-Flag antibody which detects HA- and Flag-tagged scFv-PD-L1 and scFvFc-PD-L1, respectively, or (right panel) with anti-2A peptide antibody which detects Env-scFv polyprotein (Env-scFv), unprocessed viral precursor envelop protein separated from the Env-scFv polyprotein (Env-2A), and processed viral envelop protein tagged with the 2A peptide at the C-terminus (p15E-2A). Anti-GAPDH antibody (lower left panel) was included as loading control.

We have previously reported a new RRV configuration utilizing the viral-derived “self-cleavage” 2A peptide for transgene expression [21] and demonstrated that RRV-2A configuration can tolerate transgene insertion up to 1.2 kb. In the current study, we designed two different configurations of a single-chain variable fragment (scFv) against PD-L1. One consists of scFv alone and another with the Fc from human IgG1, designated pAC3-scFv-PDL1 and pAC3-scFvFc-PDL1, respectively. Due to the absence of an antibody against scFv PD-L1 protein, we also generated a matching pair of the constructs with an HA and Flag epitope incorporated at the C-terminus of the transgene, designated pAC3-scFv-HF-PDL1 and pAC3-scFvFc-HF-PDL1 (Figure 1A).

We have also previously shown that transgenes targeted for different cellular compartments encoded in-frame with the viral envelope (Env) protein in the RRV-2A configuration, are efficiently separated from Env-transgene polyprotein [21]. Because both the epitope tagged and untagged scFv PD-L1 and scFvFc PD-L1 proteins are designed to be separated from the viral Env protein and secreted from the cells, we used a transient transfection system to highly overexpress the transgene proteins to aid the detection of epitope tagged scFv PD-L1 and scFvFc PDL1 proteins. Cell lysates from transiently transfected 293T cells were resolved on SDS-PAGE and detected with anti-HA and anti-Flag antibody to confirm the presence of scFv PD-L1 and its separation efficiency mediated by the 2A peptide, respectively. In addition, an anti-2A antibody was also included to confirm the proper processing of the viral Env protein from the polyprotein. Figure 1B shows that both scFv-HF PD-L1 and scFvFc-HF PD-L1 are detected and separated from the polyprotein as expected, and that the viral Env protein is properly processed to its subunits as indicated by the detection of 15E-2A [21]. The residual unseparated polyprotein detected is also expected as the cell lysates are from transiently transfected system in which the protein is highly overexpressed, and it was previously shown that such unseparated polyprotein is not incorporated into viral particles [21].

### RRV vectors encoding scFv-PDL1 and scFvFc-PDL1 replicate efficiently and exhibit high level of genome stability

**Figure 2:**
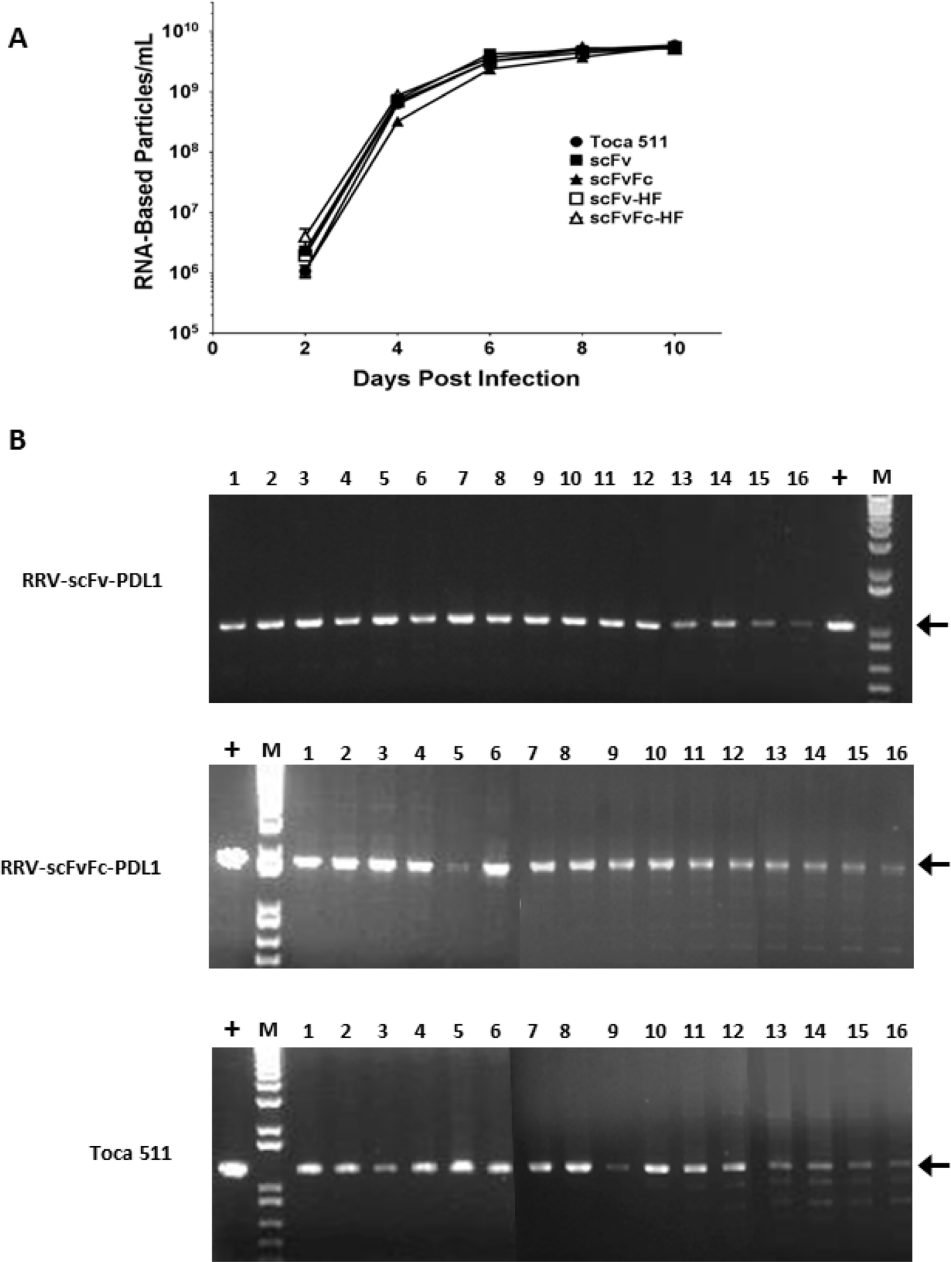
Replication kinetics and genome stability of RRV-scFv-PDL1 and RRV-scFvFc-PDL1 vectors (**A**) Viral genomes isolated from the viral supernatant of during the course of infection in U87-MG cells were quantified by RT-qPCR using primers spanning the viral envelope region. Infected cells were passaged every other day during the 10 days of infection and supernatants collected at each indicated time point prior cell passage. Data shown represent one of three independent experiments. Toca 511 (a.k.a. RRV-IRES-yCD2) was included as controls. Data shown represent one of the two independent experiments with each experiment performed in triplicates. Values represent mean ± SD. (**B**) Genome stability of RRV-scFv-PDL1 and RRV-scFvFc-PDL1 in U87-MG cells over a total of 16 infection cycles. Positive controls are PCR products amplified from plasmid DNA. Arrows indicate expected size of the PCR products. M indicates DNA molecular ladder (1 Kb Plus, Life Technologies).

We next evaluated the viral function of both epitope tagged (HA and Flag) and untagged RRV-scFv-PDL1 and RRV-scFvFc-PDL1 vectors, as proper viral Env processing is an important determinant for viral replication [24]. We first measured the viral titer produced from transiently transfected 293T cells. Our data indicate that there were no significant differences in titers produced among the four vectors from transient transfection (Supplementary Table 1). In addition, viral replication kinetics of all four vectors were monitored in a conventional human glioma cell line U87-MG, and Toca 511 was included as a control and a comparator. Figure 2A shows that with equal input of multiplicity of infection (MOI) at the initial infection, all four vectors replicated efficiently in U87-MG cells with no delay observed compared to Toca 511.

Another key factor which could influence the therapeutic effect of the transgene is vector stability. We have previously showed that the RRV-2A can tolerate a large transgene(s) insertion up to 1.2 kb [21]. In the current study, the long-term vector genome stability of RRV-scFv-PDL1 and RRV-scFvFc-PDL1 in U87-MG cells was followed for a total of 16 infection cycles. The proviral DNA from maximally infected cells at each infection cycle were harvested for PCR. Our data indicate that the viral genome of both RRV-scFv-PDL1 and RRV-scFvFc-PDL1 remained stable throughout the 16 cycles of infection (Figure 2B). Interestingly, the data also indicate that RRV-2A configuration can tolerate a transgene size of 1.6 kb (scFvFc transgene) which is 0.4 kb larger than the thymidine kinase transgene previously reported [21].

### scFv PD-L1 and scFvFc PD-L1 secreted from RRV-scFv-PDL1 and RRV-scFvFc-PDL1 infected cells competes with PD-1 for PD-L1 binding

**Figure 3:**
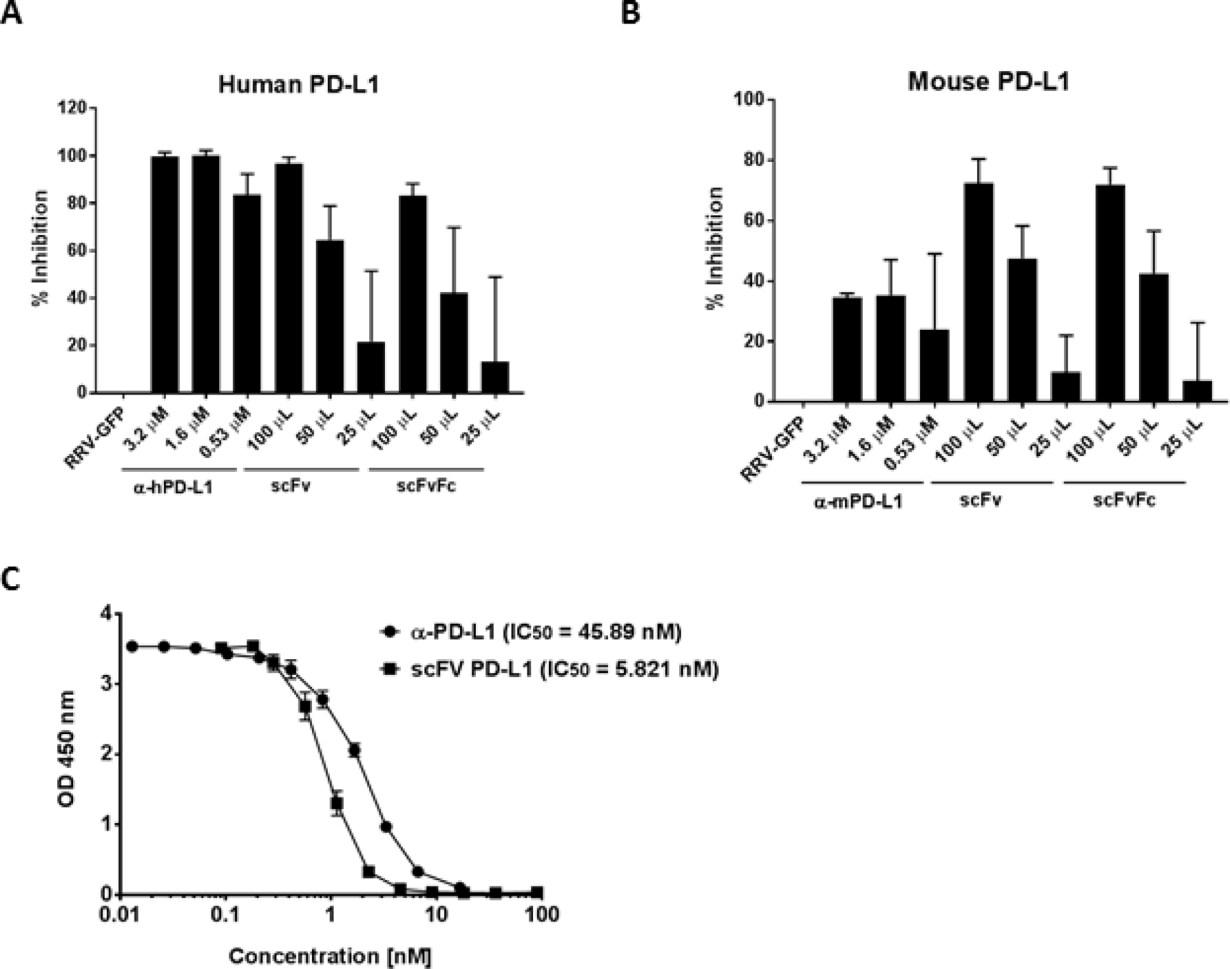
Detection of scFv PD-L1 binding to PD-L1 by competitive ELISA Wells in a 96-well microtiter plate were coated with (**A**) recombinant human or (**B**) mouse PD-L1-Fc followed by co-incubation with His-tagged recombinant PD-1-Fc in competition with 25, 50 or 100 µL of supernatant of undefined scFv PD-L1 (scFv) and scFvFc PD-L1 (scFvFc) protein concentration collected from CT26 cells maximally infected with RRV-scFv-PDL1 and RRV-scFvFc-PDL1, respectively. Anti-human or mouse PD-L1 antibody was included as positive control (indicated as α-hPD-L1 and α-mPD-L1). Anti- His tag antibody was used to detect bound His-tagged PD-1-Fc. Optical density was measured at 450 nm. The percentage of inhibition was calculated with respect to the supernatant from CT26 maximally infected with RRV-GFP (non-scFv-PD-L1) used in the competition. Error bars indicate the standard deviation of the dataset. (**C**) Wells were coated with recombinant human PDL1-Fc followed by co-incubation with His-tagged recombinant PD-1-Fc in competition with purified scFv PD-L1 or anti-human PD-L1 antibody range from 0.01–100 nM. IC50 values were determined using best-fit values from non-linear three-parameters logistic.

Having confirmed the transgene protein expression and viral function of RRV-scFv-PDL1 and RRV-scFvFc-PD-L1, we evaluated the binding characteristics of scFv PD-L1 and scFvFc PD-L1. The potency of scFv PDL1 and scFvFc PD-L1 protein to block PD-1/PD-L1 interaction was evaluated using an ELISA-based competition assay to quantify the amount of His-tagged PD1 that remained bound to PD-L1 after coincubation of recombinant PD-L1-Fc with scFv PD-L1 or scFvFc PD-L1 protein. Although the concentration of the scFv PD-L1 and scFvFc PD-L1 in the supernatant is undefined, they specifically bound to human PD-L1 and mouse PD-L1 in a dose-dependent manner. The level of inhibition using 100 µL of the supernatant was comparable to that of the blocking antibody control with no significant difference between scFv PD-L1 and scFvFc PD-L1 (Figure 3A). The potency of scFv PD-L1 and scFvFc PD-L1 in blocking mouse PD-1/PD-L1 interaction appears to be effective though slightly less potent than with the human counterpart but more effective than the anti-mouse PD-L1 antibody (Figure 3B). To further evaluate the binding activity of scFv PD-L1 on human PD-1/PD-L1, we compared the blocking activity of purified scFv PD-L1 to that of the anti-human PD-L1 antibody. Despite of its monovalent property, scFv PD-L1 exhibits an approximately 10-fold higher blocking activity (01C;than anti-human PD-L1 antibody (Figure 3C, IC50 = 5.821 nM vs. 45.89 nM). We also characterized the binding kinetics of scFv PD-L1 to human and mouse PD-L1 using the surface plasmon resonance system. The scFv PD-L1 cDNA was cloned into a CMV-driven expression vector for transient transfection followed by purification to obtain >85% purity. The equilibrium dissociation constant (K_**D**_) of scFv PD-L1 for recombinant human PD-L1 and mouse PD-L1 were determined to be 0.426 nM and 4.78 nM, respectively, (Supplementary Figure 1). The approximately 10-fold higher binding affinity to human PD-L1 as a result of slower K_off_ could explain the higher potency of scFv PD-L1 in blocking human PD-1/PD-L1 interaction observed in the competitive ELISA.

### scFv PD-L1 secreted from RRV-scFv-PDL1 infected cells exhibits bystander trans-binding activity to PD-L1 on the cell surface

As infection of 100% of patient tumor cells *in situ* is not currently feasible by any viral-based therapeutic approach including RRV, we designed a secreted transgene product with the capacity to bind PD-L1 on neighboring, uninfected cells. Here, we employed a cell-based assay to confirm antigen-specific binding of scFv PD-L1 or scFvFc PD-L1 by flow cytometry. In this experiment, we used the epitope tagged scFv PD-L1 and scFvFc PD-L1 (scFvHF PD-L1 and scFvFc-HF PD-L1) followed by anti-HA antibody for detection. The data show that scFvHF PD-L1 and scFvFc-HF PD-L1 bind to PD-L1 expressed on cell surfaces in human and mouse cell lines as indicated by a marked shift in mean fluorescent intensity (MFI) with an anti-HA antibody (Supplementary Figure 2). A higher shift in MFI observed with scFvFc-HF PD-L1 in both the human and mouse cell lines tested is likely due to bivalent dimer of scFvFc-HF PD-L1 and hence simply a reflection of more anti-HA antibody bound to scFvFc-HF PD-L1 on the cell surface, rather than increased binding affinity, as the scFvFc PD-L1 did not compete more effectively than scFv PD-L1 in the ELISA (Figure 3A and 3B). Furthermore, the antigen binding specificity was demonstrated by blocking the accessibility of an anti-PD-L1 blocking antibody to PD-L1 on the cell surface when co-incubated with the anti-HA antibody, resulting in a marked decrease in the MFI with the anti-PD-L1 antibody staining (Supplementary Figure 2). Consistent with the data observed in the competitive ELISA, scFv-HF PD-L1 and scFvFc-HF PD-L1 bind specifically to PD-L1 on the cell surface and block anti-PD-L1 antibody binding to PD-L1, suggesting the epitope for scFv-HF PD-L1 and scFvFc-HF PD-L1 overlaps or is in proximity to that of the anti-PD-L1 antibody. In addition, the marked decrease in the MFI with anti-PD-L1 antibody also suggests full receptor (PD-L1) occupancy on the cell surface.

**Figure 4:**
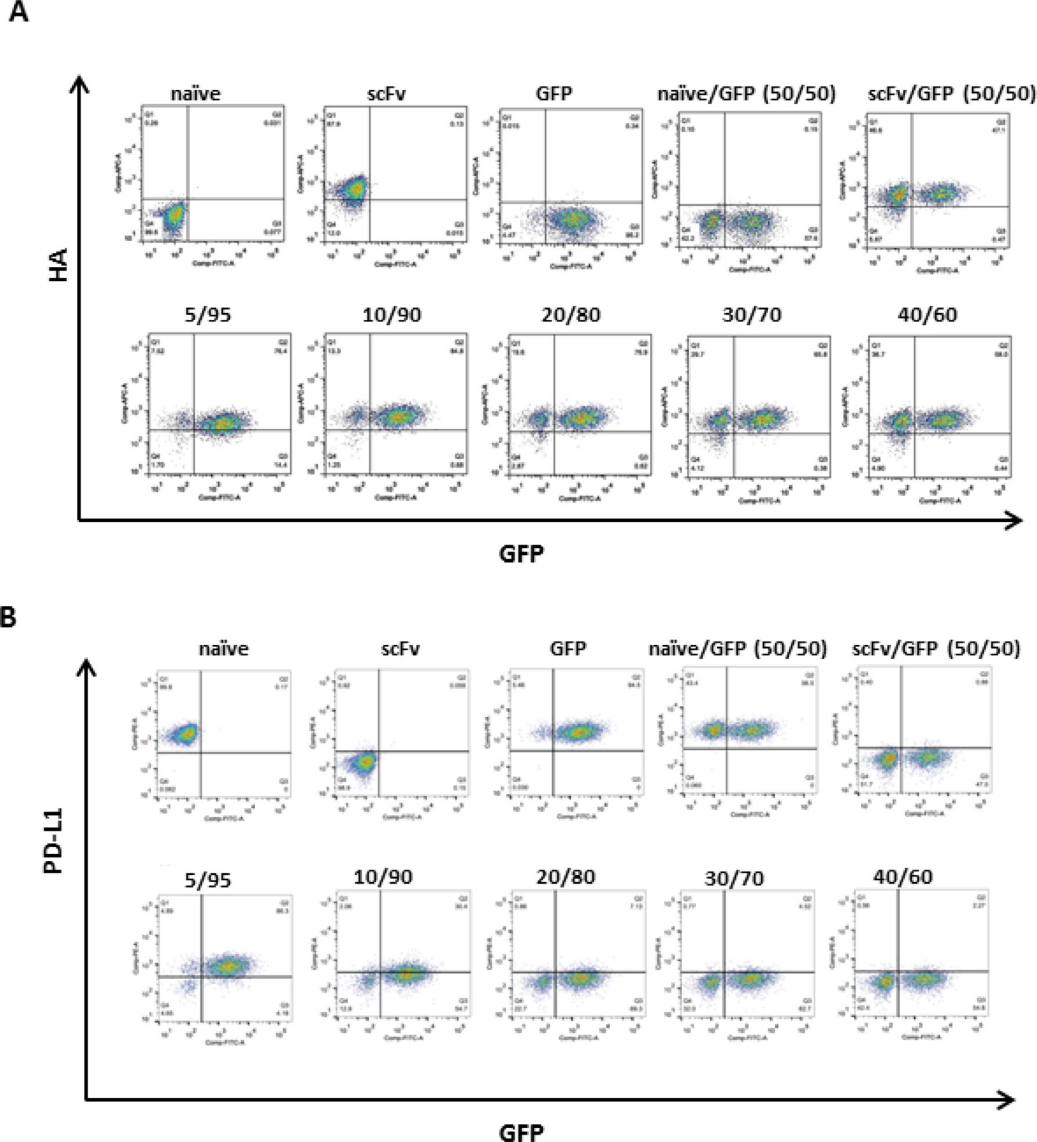
scFv PD-L1 *trans*-binding activity to PD-L1 on the cell surface of bystander cells IFNγ-treated EMT6 cells maximally infected with RRV-scFv-HF PDL1 (HA-tagged scFv-PD-L1) or RRV-GFP at ratios indicated in parenthesis were split into 2 sets. (**A**) One set of cells was stained with Alexa Fluor 647-conjugated anti-HA antibody and (**B**) the second set of cells was stained with PE-conjugated anti-mouse PD-L1 antibody. HA-positive, PD-L1-positive, and GFP-positive cell populations were measured by flow cytometric analysis.

We have previously demonstrated in tumor mouse models that approximately 10–15% transduction level of Toca 511 in the tumor prior to initiation of 5-FC treatment is required to achieve long-term survival benefit through an immune-mediated bystander effect, and a minimal of at least 2–3% transduction to achieve some therapeutic benefit [2, 21]. To evaluate the bystander effect of RRV-scFv-PD-L1 *in vitro*, we tested the minimal transduction level required to achieve full receptor occupancy on tumor cells. In this experiment, EMT6 mouse breast cancer cells maximally infected with RRV-scFv-HF-PDL1, mixed with EMT6 cells maximally infected with RRV-GFP at various ratios were co-cultured to measure bound scFv-HF PD-L1 and unbound PD-L1 on the cell surface using the anti-HA and anti-PD-L1 antibody. Our data show that bound scFvHF PD-L1 was detected on all cell surfaces when only 5% of the cells express scFvHF PD-L1 (Figure 4A). The full occupancy of PD-L1 inversely correlates with the decrease in PD-L1 signal on the cell surface in a dose dependent manner (Figure 4B), suggesting that scFv PD-L1 can achieve 100% bystander effect with a minimal level of transduction.

### scFv PD-L1 rescues PD-1/PD-L1 mediated immune suppression *in vitro*

**Figure 5:**
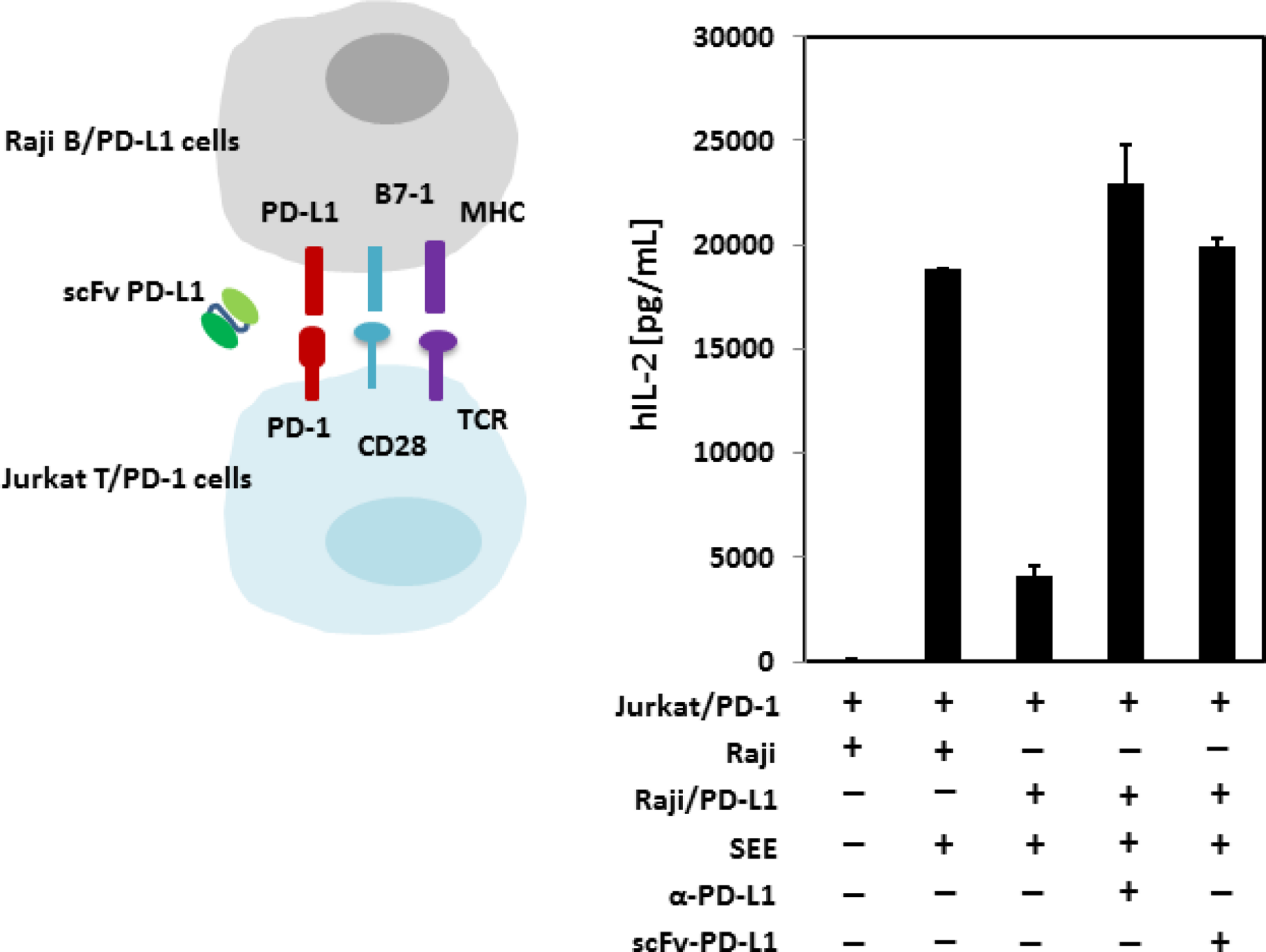
*In vitro* immune function of scFv PD-L1 Mixtures of SEE loaded, SEE + anti-human PD-L1 (10 µg/mL) or SEE + purified scFv-PD-L1 (10 µg/mL) Raji B cells or Raji B cells overexpressing human PD-L1 (with rested Jurkat T cells overexpressing human PD-1) were co-cultured in RPMI-1640 medium containing 10% human AB serum for stimulation for 20–22 hours followed by IL-2 measurement by ELISA.

As we have demonstrated that scFv PD-L1 binds to human PD-L1 with sub-nanomolar affinity and competes effectively with PD-1 for PD-L1 binding, we asked whether the blockade of PD-1/PD-L1 interaction in a cell-based system leading to immune suppression can be rescued by scFv PD-L1. In this experiment, we used an *in vitro* co-culture system previously described to demonstrate PD-1/PD-L1 mediated T-cell immune suppression [25]. The system was first validated to confirm that all signaling components for stimulation are present on Jurkat T-cells and Raji B-cells to respond to stimulation by the super antigen staphylococcal enterotoxin E (SEE), (Supplementary Figure 3A and 3B). We then generated Jurkat T cells and Raji B cells overexpressing human PD-1 and human PD-L1, respectively, by lentiviral vector transduction (Supplementary Figure 3C). Consistent with reported data, stimulation by SEE in the absence of PD-1/PD-L1 engagement led to a large quantity of IL-2 production by the Jurkat T cells, whereas PD-1/PD-L1 engagement led to a >75% decrease in IL-2 production as a phenotypic indication of immune suppression (Figure 5). Importantly, in agreement with the binding characterization data described above, scFv PD-L1 effectively blocked PD-1 for PD-L1 engagement and rescued the T-cell immune suppression mediated by PD-1/PD-L1.

### scFv PD-L1 and scFvFc PD-L1 treatment lead to tumor growth inhibition in a dose dependent manner and elicit immune memory response in syngeneic tumor models

To translate the bystander effect and immune function of scFv PD-L1 observed *in vitro* to anti-tumor activity *in vivo*, we first sought to evaluate the anti-tumor immune activity mediated by scFv PD-L1 and scFvFc PD-L1 in a CT26 mouse model which is commonly used for testing the efficacy of checkpoint inhibitors with marginal anti-tumor activity when administered as a monotherapy [26, 27]. In this experiment, increasing percentages of CT26 cells pre-transduced with RRV-scFv-PDL1 or RRV-scFvFc-PDL1 vector were prepared to evaluate survival with viral spread occurring during tumor growth. Tumor implants of RRV pre-transduced at 2%, 30% and 100% expressing scFv PD-L1 or scFvFc PD-L1 were assessed for survival over time in comparison to untreated tumor-bearing mice. The results indicate that the hazard ratios for tumor expressing scFv PD-L1 or scFvFc PD-L1 range from 0.384 to 0.7 relative to that of untreated mice (Supplementary Figure 4). For tumors that expressed scFv PD-L1, as low as 2% pre-transduced tumor was sufficient to provide survival benefit and the expression of IgG1 Fc as part of the scFvFc PD-L1 intended for improving protein stability or biodistribution of scFvFc PD-L1 appears to be dispensable as it did not improve survival benefit over scFv PD-L1 expressing tumors in all 3 ratios tested. Notably, anti-PD-1 blocking antibody treatment, with dosing in a pre-staged tumor setting, did not provide survival benefit.

We have shown that *in vitro* scFv PD-L1 secreted from as low as 5% pre-transduced cells exhibited bystander *trans*-binding activity, leading to a full PD-L1 occupancy on the cell surface of non-scFv PD-L1 expressing cells. We next evaluated the dose response of the anti-tumor activity of scFv PD-L1 in a syngeneic orthotopic EMT6 breast cancer model which has been reported to be responsive to checkpoint inhibitors [28, 29]. To evaluate the anti-tumor activity of scFv PD-L1 and scFvFc PD-L1 in a more clinically relevant scenario, we sought to determine the minimal transduction level required for scFv PD-L1 to achieve anti-tumor activity, using different ratios of EMT6 cells maximally pre-transduced with RRV-scFv-PDL1, RRV-scFvFc-PDL1 or RRV-GFP vectors. These cells are resistant to further RRV infection mediated via the amphotropic envelope protein due to receptor down regulation [21]. The data show that mice bearing tumors with 2%, 30% and 100% scFv PD-L1 or scFvFc PD-L1 expression trend toward a survival benefit compared to untreated mice, and a statistical significance was observed between the untreated and 30% scFvFc/scFvFc group (Supplementary Figure 5A). We further sought to investigate whether mice that survived from the primary tumor have established an anti-tumor immune memory response by re-challenging them with naïve EMT6 tumor cells on the flank. Data revealed that mice that cleared tumor with scFv/scFvFc treatment in the primary setting exhibited a moderate delayed tumor growth in a re-challenge setting, suggesting that an anti-tumor immune response was established in these mice (Supplementary Figure 5B). Together, these data indicate that tumor cells expressing scFv PD-L1 or scFvFc PD-L1 can lead to anti-tumor activity that appears to be superior to treatment with an anti-mouse PD-1 antibody.

**Figure 6:**
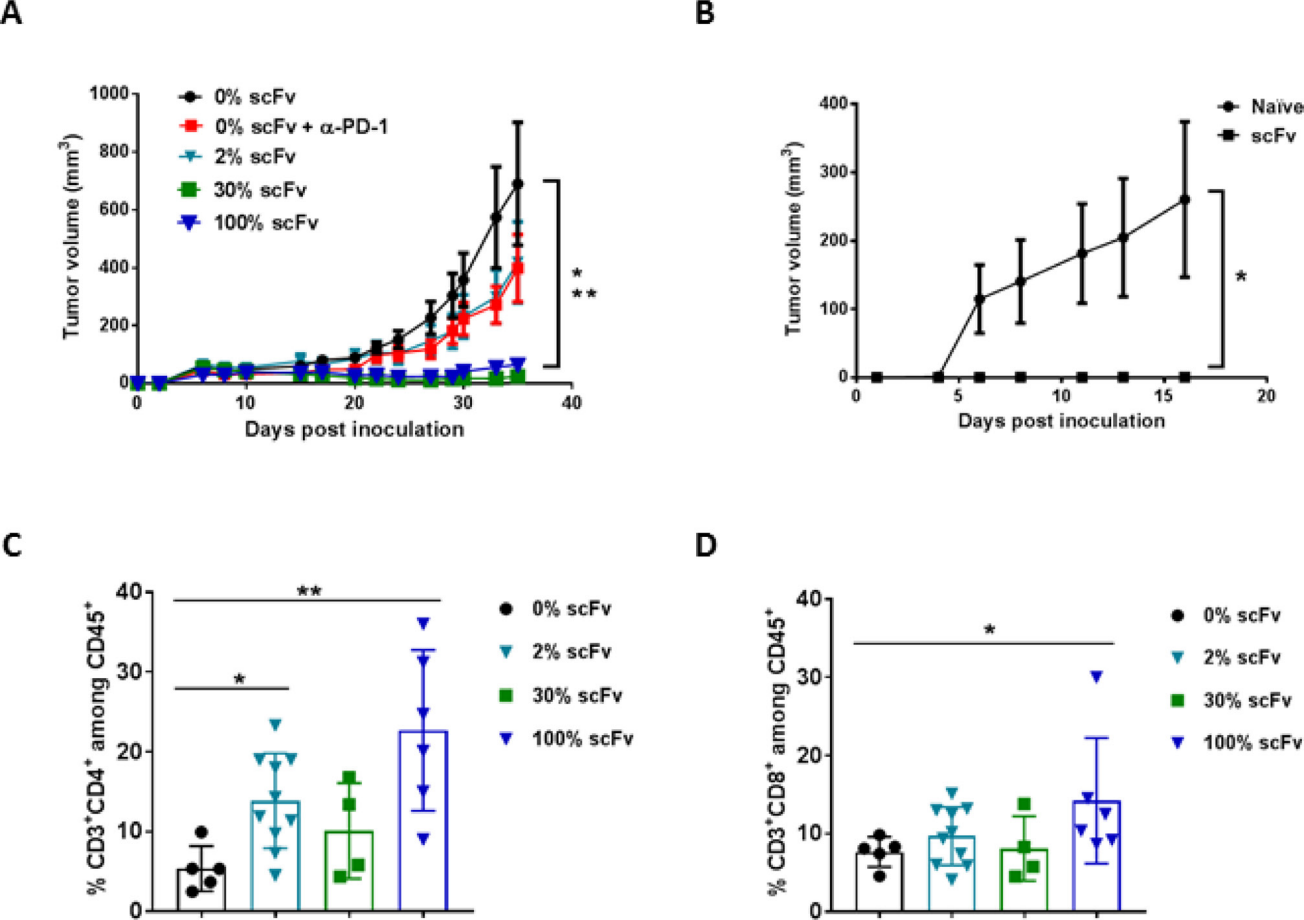
Pre-transduced tumor cells expressing scFv PD-L1 demonstrate a dose-dependent anti-tumor activity and enhanced tumor infiltrating lymphocytes (**A**) Subcutaneous tumor model using Tu-2449SC cells maximally infected with RRV-scFv PDL1 or RRV-GFP at indicated ratios were subcutaneously implanted on the right flank of 8-week-old female B6C3F1 mice and assigned to indicated groups (*n* = 10 per group). Control groups are mice implanted with 100% Tu-2449SC/RRVGFP cells that received PBS (0% scFv) or anti-mouse PD-1 antibody (0% scFv + α-PD-1). Anti-PD-1 antibody (Clone J43) was i. p. administered on day 0 (300 µg per mouse), day 3, day 6 and day 9 (200 µg per mouse). Statistical significance was determined by 2-way ANOVA of the following data sets: ^*^p < 0.0001 for 0% vs 30% scFv; ^**^p < 0.0001 for 0% vs 100% scFv. (**B**) Naïve mice (*n* = 5) and mice that cleared their initial tumor implant from RRV-scFv-PDL1 treated group (*n* = 15) were challenged with 2 × 10^6^ Tu-2449SC on the left side of the flank. Tumor growth and measurement were monitored over time. ^*^p < 0.0001 (0% scFv vs. 2%, 30% and 100% scFv). Error bars indicate the standard error measurement (SEM) of the dataset. (**C**) and (**D**) Frequency of tumor-infiltrating CD4^+^ (C) and CD8^+^ (D) lymphocytes among CD45^+^ cells by flow cytometry, 39 days post-tumor implantation. Statistical analyses were performed using Mann-Whitney test at 95% CI (^*^P < 0.05, ^**^P < 0.01). Values represent means ± SD of the dataset.

We have previously reported on a subcutaneous Tu2449SC tumor model in B6C3F1 mice to elucidate the immune-mediated mechanism of action of Toca 511 and Toca FC [2]. We also tested this tumor model to determine the minimal transduction level required for scFv PD-L1 to exert anti-tumor activity. Consistent with data from the two tumor models described above, Figure 6A shows that in the Tu-2449SC tumor model, mice bearing tumor with as low as 2% Tu-2449SC cells expressing scFv PD-L1 led to a delay in tumor progression that is comparable to anti-PD-1 antibody treatment, but not statistically significant when compared to untreated mice (Figure 6A). With 30% pre-transduced cells, tumor progression was strongly inhibited as also seen in mice bearing tumors with the 100% pre-transduced cells. In this model, mice that cleared tumor with scFv treatment in the primary setting exhibited a strong memory anti-tumor response in a re-challenge setting (Figure 6B). Expectedly, the benefit of Fc in scFvFc PD-L1 was again not observed in this tumor model. Intriguingly, the inclusion of Fc at higher ratios (30% and 100% scFvFc PD-L1) seems to reduce its anti-tumor activity (Supplementary Figure 6).

To demonstrate PD-1/L1 blockade is associated with enhanced tumor lymphocyte infiltration, in a separate experiment, mice from each assigned group that had measurable tumor growth were extracted at day 39 post tumor implantation for lymphocyte profiling. The data show that treatment with scFv PD-L1 resulted in a modest increase in CD8+ and a significant increase in CD4+ cells across all 3 groups when compared to the control group which have tumors with 100% RRV-GFP cells (Figure 6C and 6D).

To further demonstrate that the anti-tumor activity of scFv PD-L1 is immune-mediated and requires functional T-cells, a comparison of Tu-2449SC tumor progression was conducted in immune competent B6C3F1 mice and in an immune-deficient athymic mouse which lack functional T cells. As expected, Tu-2449SC tumor growth was delayed in B6C3F1 mice, whereas in athymic mice, Tu-2449SC tumor growth progressed without delay (Supplementary Figure 7).

### Intracranial injection of RRV-scFv-PDL1 prolongs survival in syngeneic orthotopic glioma model

**Figure 7:**
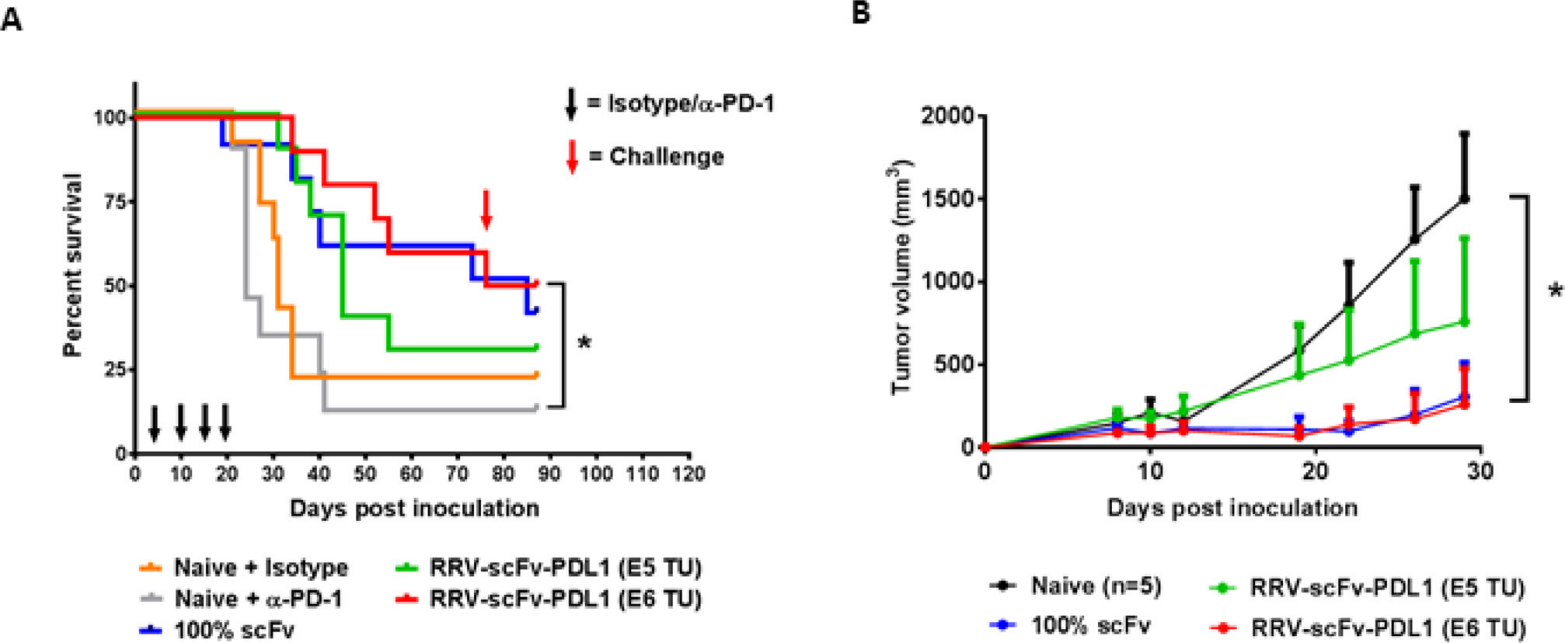
Orthotopic glioma model with intracranial injection of RRV-scFv-PDL1 demonstrates a dose-dependent anti-tumor activity (**A**) Female B6C3F1 mice (8-week-old; *n* = 10 per group) were i.c. implanted with 1 × 10^4^ of Tu-2449 cells. Survival analysis was monitored for 90 days. Mice in the experimental groups were injected with 1E5 or 1E6 TU of purified RRV-scFv-PDL1 vector on day 4 post tumor implant. Control groups are mice bearing 100% pre-transduced scFv PD-L1 expressing tumor cells (100% scFv) and mice treated anti-PD-1 antibody (Naive + αPD-1) or isotype control (Naive + Isotype), (300 µg per mouse i.p. induction on day 4; 200 µg per mouse maintenance dose on day 10, 14 and 17 indicated by black arrows). Survival data were plotted by the Kaplan–Meier method. Statistical significance of survival between mice treated with anti-PD-1 antibody and injection-treated RRVscFvPDL1 mice was determined by the Log-rank (Mantel-Cox) test, ^*^p **=** 0.0045. (**B**) Mice which had survived from initial tumor implant from RRV-scFv-PDL1 pre-transduced or injectiontreated groups were challenged with 2 × 10^6^ Tu-2449SC cells on the right flank on day 80 (red arrow) post primary tumor implant. Tumor growth and measurement were monitored over time. Statistical significance was determined by 2-way ANOVA of the following data sets: ^*^p < 0.0001 for Naive vs 1E6 TU RRV-scFv PD-L1. Error bars indicate the SEM of the dataset.

The intracranial anti-tumor activity of immune checkpoint inhibitors have been clinically investigated recently in patients with recurrent glioblastoma (GBM) and preliminary data demonstrated a failure of anti-PD-1 treatment as a monotherapy to prolong survival (Checkmate 143, NCT 02017717) [30, 31]. Despite the fact that a significant number of tumors expressed PD-L1 in GBM [32], the pre-clinical evidence supporting the anti-tumor activity of anti-PD-1/PD-L1 has been limited to one orthotopic syngeneic glioma model [33–35]. Thus, we investigated scFv PD-L1 anti-tumor activity in an orthotopic syngeneic glioma model previously reported to respond to Toca 511 and Toca FC treatment [3] and employed an intra-tumoral RRV delivery approach previously established [3]. RRV-scFv-PDL1 viral functions and genome stability in maximally infected Tu-2449 cells were confirmed *in vitro* (Supplementary Figure 8). In this experiment, two different doses of RRV-scFv-PDL1 (1E5 and 1E6 TU) were delivered by a single intra-tumoral injection 4 days after tumor implant. Our data show that a single administration of 1E6 TU of RRV-scFv-PDL1 is equally effective as Tu-2449 cells maximally pre-transduced with RRV-scFv-PDL1 (Figure 7A). Further, consistent with observation made in the previous experiments, subcutaneous re-challenge of Tu-2449SC tumor cells at a remote site from the primary tumor showed a systemic anti-tumor immune response leading to significant delay in tumor growth compared to naïve mice (Figure 7B). Moreover, we also demonstrated that GL-261 tumor cells infected with RRV-scFv-PDL1 exhibited anti-tumor activity in a syngeneic orthotopic glioma model in C57BL/6 mice (Supplementary Figure 9). Together, these findings indicate that scFv PD-L1 has anti-tumor activity in two glioma tumor models and Tu-2449 tumor model represents a previously undescribed glioma mouse model that responds to checkpoint inhibitors as a monotherapy.

### RRV-scFv-PDL1 infected tumors do not secrete appreciable levels of scFv PD-L1 into the systemic circulation

**Figure 8:**
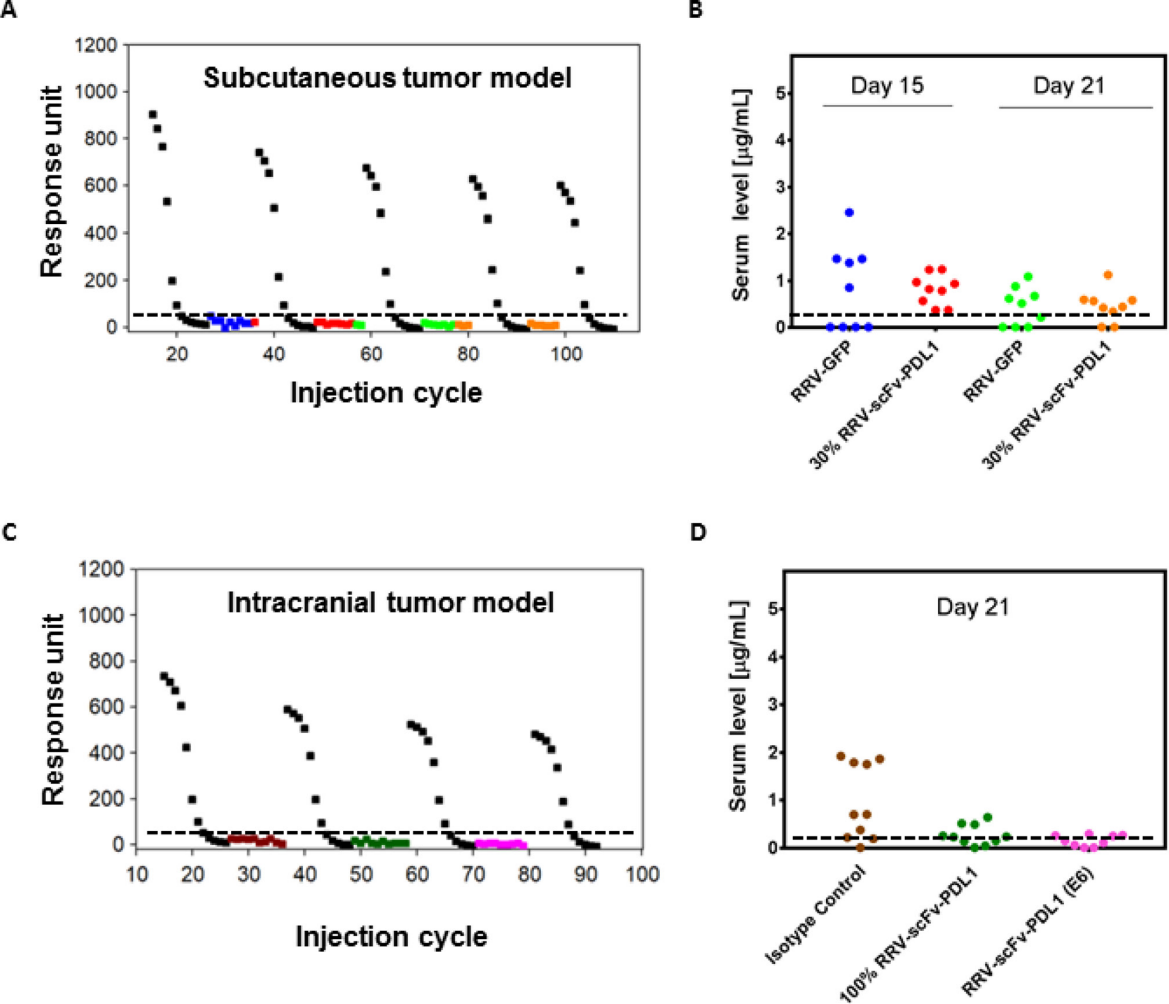
scFv PD-L1 serum levels in tumor-bearing mice (**A**, **B**) Sera from Tu2449 subcutaneous tumor pre-transduced with 30% RRV-scFv-PDL1 and (**C**, **D**) Tu-2449 intracranial tumor treated with isotype, pre-transduced with 100% RRV-scFv-PDL1 or injected with 1 × 10^6^ TU of RRV-scFv-PDL1 were collected at indicated time point post tumor implant. scFv PD-L1 serum concentrations were measured by surface plasmon resonance with standard curves at concentrations ranging between 0.1 nM–200 nM (0.0028 µg/mL-5.6 µg/mL) using naïve serum spiked-in with purified scFv PD-L1 and serve as positive controls. Sera from RRV-GFP pre-transduced tumor or isotype antibody treated tumor-bearing mice were included for baseline levels. Each symbol represents one mouse serum sample. Each colored symbol except black represents one mouse serum sample. Black, standard curves generated for every 20 cycles of regeneration. (B) and (D) Graphical presentation of the data conversion from responsive unit shown in (A) and (C) to µg/mL. The lower limit of quantification (LLOQ) is determined to be 0.011 µg/mL (0.39 nM) and the dashed horizontal line indicates LLOQ of the assay. For graphical presentation, samples measured below LLOQ were entered with a fixed value of 0.010 µg/mL. The data represent one data set from two separate experiments.

Since RRV-scFv-PDL1 is delivered as a virus rather than a therapeutic antibody and that scFv PD-L1 is produced from the infected tumor cells, scFv PD-L1 serum level is anticipated to be significantly lower than the levels reported for therapeutic antibodies delivered intravenously. Given the possibility that scFv PD-L1 may distribute into circulation during tumor lysis, we collected serum from tumor-bearing mice in both subcutaneous and intracranial models that demonstrated anti-tumor activity and measured scFv PD-L1 using a surface plasma resonance method. As expected, our data reveal that the scFv PD-L1 serum concentrations from tumor-bearing mice expressing scFv PD-L1 were comparable to that of negative control groups in both models (Figure 8A–8D). Although most of the samples measured had signals below the lower limit of quantification (LLOQ), some, particularly from the subcutaneous tumor model, were measured at background level above LLOQ. As these values were observed in mice from the negative control group (100% RRV-GFP tumor or tumor treated with isotype antibody), we assume it has no significance and represents a non-specific signal from sample components. These findings indicate that scFv PD-L1 expressing tumor does not lead to prolonged exposure of the protein in the serum, presumably due to a combination of local production, high level of tumor retention, and/or short lifespan of the protein in the absence of an Fc region. In all cases, the nominal detected scFv PD-L1 serum concentration or presumably a non-specific signal is at least 50 to 150-fold less than the levels reported for anti-PD-1/PD-L1 therapeutic antibodies approved for various clinical indications.

## DISCUSSION

Checkpoint inhibitor blockade has proven to be a promising treatment across many cancer types. However, they are not always effective and are associated with systemic immune toxicities which can lead to discontinuation of treatment. In this study, as a proof of principle, we have demonstrated that PD-1/PD-L1 mediated immune suppression can be rescued by RRV delivery of a secreted scFv targeting PD-L1 in tumors and immune infiltrating cells in the TME resulting in increased survival and a protective anti-tumor memory response associated with minimal serum exposure of scFv PD-L1. Most importantly, in contrast to oncolytic virus, the anti-tumor activity observed in tumor-bearing mice treated with RRV-scFv-PDL1 in our studies is not associated with or primed by innate antiviral immunity as shown by 100% RRV-GFP infected tumor’s growth kinetics (included as one of the control groups rather than naïve tumors).

The advantages of using tumor-selective RRV for delivering scFv PD-L1 in the TME are at least two fold. One is the localized and sustained delivery of therapeutic payload in the TME with relatively short half-life compared to recombinant therapeutic antibodies. These features allow for engagement of the checkpoint to promote anti-tumor activity in TMEs that may be difficult to reach with conventional systemic delivery of CPI monoclonal antibodies and consequent efficacy in otherwise poorly responsive tumors as well as avoidance of undesirable binding to healthy tissue leading to systemic immune toxicity [36]. Another advantage may be the prevention of developing an anti-drug antibody response due to repeated systemic infusion of the biotherapeutic [37, 38]. Innovative approaches to circumvent autoimmune toxicity are currently being evaluated. CX-072, an engineered antibody designed for localized therapeutic activity through reliance on tumor-expressing protease(s) to unmask the antigen binding domain targeting PD-L1 in the TME recently reported a favorable safety profile (http://ir.cytomx.com/news-releases/news-release-details/cytomx-therapeutics-presents-preliminary-clinical-proof-concept). However and notably, grade 3/4 immune-related toxicity was still identified in some patients.

We and others have previously demonstrated PD-1/PD-L1 blockade using the RNAi and aptamer approach [39–41]. Nevertheless, limitations of these approaches are related to tumor selectivity and/or lack of a bystander effect. By contrast, overexpression of tumor-selective scFv PD-L1 protein by less than 10% RRV-scFv-PDL1 infected cells *in vitro* can fully occupy PD-L1 on bystander cells which translates to a high level of anti-tumor activity in several mouse tumor models. Also, RRV-scFv-PDL1 efficacy was evaluated in several syngeneic mouse tumor models in pre-transduced setting to evaluate dose effect and via intratumoral vector injection to mimic a potential clinical setting. A dose-dependent anti-tumor activity was observed in all three tumor models tested, indicating that 2% transduction level led to similar therapeutic effect as the anti-PD-1 antibody treated group and that a higher transduction level demonstrated anti-tumor activity that is superior than anti-PD-1 antibody group, suggesting the level of PD-L1 occupancy by anti-PD-1 antibody infusion may be suboptimal at the tumor site.

Given that RRV has been demonstrated to be cancer-selective both in preclinical and clinical settings [3, 42], an enhanced anti-tumor activity owing to longer half-life than scFv PD-L1 and/or potential antibody-dependent cell-mediated cytotoxicity was not observed in our pre-transduced tumor models. To the contrary, the presence of Fc in scFvFc PD-L1 appears to diminish anti-tumor activity which is most evident in the Tu-2449SC tumor model (Supplemental Figure 6). A possible explanation of this phenomenon is suggested by a recent report which revealed that bound anti-PD-1 antibody on T cells can be phagocytosed by PD-1-negative tumor associated macrophages via interaction between the antibody Fc glycan and FcγR on the macrophages, limiting antibody retention in the TME and reducing antibody-induced anti-tumor activity [43]. Importantly, scFv PD-L1 anti-tumor activity is associated with serum levels below the limit of quantification which is at least 50- to 150-fold less than most CPI antibodies levels reported in patients administered antibody systemically (Figure 8). Furthermore, along with proximal target engagement within the TME, a significant, higher binding affinity of scFv PD-L1 to human PD-L1 than human PD-1 to PD-L1 (K_D_ = 0.426 nM vs 0.77 µM) [16] as well as a significantly longer half-life of scFv PD-L1 bound to human PD-L1 (Supplementary Figure 1), (t_1/2_ = ∼77 min for scFv PD-L1/PD-1 vs. 0.9 sec for human PD-L1/PD-1) [44] may provide therapeutic benefits for the RRV TME-centered CPI immunotherapy.

Immunotherapy studies in brain tumor have been limited by the lack of representative murine models. The anti-tumor activity observed in the Tu-2449 syngeneic orthotopic glioma model brings a second model other than GL-261 reported to respond to checkpoint inhibitor as a monotherapy [34, 35, 45, 46]. The Tu-2449 tumor has been described to closely resemble the development of human glioblastoma with characteristic of hyper-vascularization [47, 48]. Despite the fact the Tu-2449 cells are immunogenic and express MHC on their cell surface (data not shown), unlike GL-261 cells which are reported to carry high tumor mutational burden (Genoud *et al.*, OncoImmunology, https://doi.org/10.1080/2162402X.2018.1501137), their tumorigenic property is not induced by carcinogen and thus are most likely to have low tumor mutational burden which closely resembles human GBM.

RRV-scFv-PDL1 could potentially be applicable in a neoadjuvant setting prior to tumor resection; this type of use with nivolumab, was recently reported to induce expansion of antigen-specific clones in lung cancer [49]. In addition, the non-essential requirement of Fc in our tumor-selective RRV delivery platform would allow inclusion of additional TME-targeted therapeutic transgene(s) in the RRV to further alleviate the immunosuppressive TME. For example a treatment leading to tumor lysis in turn leads to dendritic cell recruitment and activation [46, 50, 51] in combination with removal of T-cell anergy by RRV-scFv-PDL1, could be beneficial. Alternatively, combination therapy of RRV-scFv-PDL1 and therapeutics with very narrow therapeutic indices such as anti-CTLA4 antibody could lead to synergistic anti-tumor activity while reducing immune toxicities reported in current combination therapies.

In conclusion, our study demonstrates that RRV-scFv-PDL1 checkpoint inhibition with a tumor-selective delivery and a high bystander index localized within the TME provides an attractive treatment regimen to target immune checkpoints.

## MATERIALS AND METHODS

### Construction of RRV-scFv-PDL1 plasmid DNAs

Two pairs of two different configurations of single-chain variable fragment (scFv) against PD-L1 were designed. One pair consists of scFv with and without the Fc from human IgG1, designated scFv-PDL1 and scFvFc-PDL1, respectively. The other pair consists of scFv-PDL1 and scFvFc-PDL1 with HA and Flag epitope incorporated at the C-terminus, designated scFv-HF-PDL1 and scFvFc-HF-PDL1. The coding sequence of each configuration contains the 3′ coding sequence of the viral envelope gene followed by the gT2A peptide sequence [21] and was synthesized with Asc I and Not I restriction sites for subcloning into pAC3-gT2A-yCD2 at the corresponding sites to replace the gT2A-yCD2 transgene cassette resulting in pAC3-scFv-PDL1, pAC3-scFvFc-PDL1, pAC3-scFv- HF-PDL1, and pAC3-scFvFc-HF-PDL1.

### Cell culture

Human 293T cells (obtained through a material transfer agreement with the Indiana University Vector Production Facility, and Stanford University deposited the cells with ATCC, SD-3515; lot 2634366), human glioma cell line U87-MG (ATCC, HTB-14), mouse colorectal cancer cell line CT26 (ATCC, CRL-2638), mouse breast cancer cell line EMT6 (ATCC, CRL-2755), mouse glioma cell line Tu-2449 [3] and Tu-2449SC [2] were cultured in complete Dulbecco’s modified Eagle’s medium. Human leukemic T cell line Jurkat Clone E6-1 (ATCC, TIB-152) and human Burkitt’s B lymphoma cell line Raji (ATCC, CCL86) were cultured in complete RPMI-1640 medium. All completed medium contains 10% fetal bovine serum (HyClone), sodium pyruvate (Cellgro), Glutamax and penicillin-streptomycin (Invitrogen).

Jurkat T cells and Raji B cells overexpressing human PD-1 (Jurkat/PD-1) and PD-L1(Raji/PD-L1), respectively, were generated by transducing the cells with lentiviral vectors coding human PD-1 or PD-L1 at MOI of 1 followed by puromycin selection (1.6 μg/mL) 2 days post transduction. PD-1 expressing Jurkat T cells and PD-L1 expressing Raji B cells were enriched under puromycin selection for 14 days and confirmed by cells surface staining with anti-PD-1 and anti-PD-L1, respectively.

### Virus production from 293T cells and virus titer

Twenty micrograms of plasmid DNA encoding the RRVs were used for transient transfection using the calcium phosphate method as previously described [40]. The viral titers of all RRVs were determined by quantitative PCR (qPCR) and viral titers, reported in transduction units per milliliter (TU/mL) [5, 22].

### Virus replication kinetics of RRVs in U87-MG cells and vector genome stability

For viral replication kinetics, 20 μL of collected supernatant from each time points during the course of infection were obtained to extract viral RNA and measure particle titer as described [40]. For vector genome stability, genomic DNA from each infection cycle was extracted from maximally infected U87-MG cells using the Maxwell 16 Cell DNA Purification Kit (Promega # AS1020) as described [22].

### Immunoblots

For the assessment of the transgene protein expression, separation efficiency of the polyproteins, and viral envelop protein processing, cells lysates were prepared as described [21].

### scFv PD-L1 bystander *trans*-binding activity to PD-L1 on the cell surface

EMT6 cells maximally infected with RRV-scFv-HF-PDL1 (HA-tagged scFv-PD-L1) or RRV-GFP at 90% confluency were treated with recombinant human IFNγ at 250 IU/mL for 24 hours to upregulate PD-L1 expression on the cell surface. One million of IFNγ-treated EMT6 cells maximally infected with RRVscFv-HF-PDL1 or RRV-GFP at indicated ratios were split into 2 sets. One set of cells was stained with Alexa Fluor 647-conjugated anti-HA antibody (BioLegend, # 682404) and the second set of cells was stained with PE-conjugated anti-human PD-L1 antibody (ebioscience, # 12-5983). HA-positive, PD-L1-positive, and GFP-positive cell populations were measured by flow cytometric analysis (FACS Canto, BD Biosciences). HA-positive vs GFP positive cell populations and PD-L1-positive vs GFP-positive cell population were calculated by 2-color compensation for proper gating (BD FACSDiva software, BD Biosciences). Cytometry data analysis was performed using the FlowJo v10 software (FlowJo LCC).

### Detection of scFv PD-L1 binding to PD-L1 by competitive ELISA

Wells in a 96-well microtiter plate were coated with 100 μL of 1.5 µg/mL recombinant human PD-L1-Fc (R & D Systems, # 156-B7) or mouse PD-L1-Fc (BioLegend, # 758206) overnight at 4°C, followed by 2 hour blocking at room temperature with blocking buffer (1X PBS + 10%FBS + 0.05% Tween 20) the next day. Subsequently, PD-L1-Fc coated wells were co-incubated with 1 µg/mL His-tagged recombinant human PD-1 (SinoBiological, # 10377-H08H) or His-tagged recombinant mouse PD-1 (abcam, # ab180051) in competition with supernatant of undefined protein concentration collected from CT26 cells maximally infected with RRV-scFv-PDL1 or RRV-scFvFc-PDL1 (100, 50 and 25 μL), anti-PDL1 antibody (anti-human PD-L1, ebioscience # 14-5983-82; anti-mouse PD-L1 BioLegend # 124302) or purified scFv PDL1 protein (Accelagen Inc.) for 2 hours at room temperature. For interaction between scFv PD-L1 and human PD-L1, bound His-tagged recombinant human PD-1 was detected using an HRP-conjugated anti-6X His tag antibody (Invitrogen, # PA1-23024). For binding interaction between scFv PD-L1 and mouse PD-L1, bound His-tagged recombinant mouse PD-1 was detected by performing an additional signal amplification step using an biotin-conjugated anti-6X His tag antibody (1:3,000), (abcam, # ab27025) followed by HRP-conjugated streptavidin (1:10,000), (abcam, # ab7403). Color development was performed by adding 3,3′,5,5′-Tetramethylbenzidine (TMB) substrate solution (Southern Biotech, # 0410-01) and terminated by TMB STOP solution (Southern Biotech, # 0412-01) after 6–10 minutes incubation. Optical density was read by a plate reader (Molecular Devices iD5) at 450 nm (with 570 nm correction) using the Soft Max Pro software. The percentage of inhibition was calculated with respect to a positive control which supernatant from CT26 maximally infected with RRV-GFP (non-scFv-PD-L1) was used in the competition.

### *In vitro* immune function of scFv PD-L1 and IL-2 ELISA

Jurkat T cells were washed and rested in serum-free RPMI-1640 medium at 37° C for 3 hours at 5 × 10^5^ cells/0.5 mL. Raji B cells were washed and resuspended in serum-free RPMI-1640 medium at 1 × 10^6^ cells/0.5 mL followed by addition of super antigen SEE (10 μg/mL, final concentration), (Toxin Technology #ET404) or SEE plus anti-human PD-L1 or SEE plus purified scFv PD-L1 (10 μg/mL) followed by incubation at 37° C for 1 hour. At the completion of incubation, SEE loaded Raji B cells were washed in serum-free RPMI-1640 medium and resuspended in RPMI-1640 medium containing 20% human AB serum (Sigma-Aldrich, # H4522) at 5 × 10^5^ cells/0.5 mL. Both rested Jurkat T cells and SEE-loaded Raji B cells were then co-cultured with equal volumes to reach 1 × 10^6^ cells/mL/well in a non-coated 24-well plate and incubated at 37° C for stimulation.

For IL-2 ELISA, the supernatants of the co-culture cells were collected 20–22 hours after stimulation and separated from the cells by centrifugation at 300 × g for 5 minutes. IL-2 production was measured by using the Quantikine ELISA Kit (R & D System, # D2050) according to the manufacturer’s protocol.

### Mouse tumor models

All animal protocols and experiments were approved by the IRB/IACUC of Explora BioLabs (San Diego, CA, USA). For the Tu-2449SC subcutaneous tumor model, 2 × 10^6^ of Tu-2449SC cells maximally infected with RRVscFv-PDL1 or RRV-GFP at indicated ratios were subcutaneously implanted on the right flank of 8-week-old female B6C3F1 mice (Harlan Sprague Dawley Inc.) and assigned to 8 groups (*n* = 10 per group). Control Group 1 and 2 which are mice implanted with 100% Tu-2449SC/RRV-GFP cells received PBS or anti-PD-1 antibody. Anti-PD-1 antibody (Clone J43) was intraperitoneally (i. p.) administered on day 0 (300 µg per mouse), day 3, day 6 and day 9 (200 µg per mouse). Group 3, 4, and 5 received ratios of 3/97, 30/70 and 100/0 of Tu-2449SC/RRV-scFv-PDL1 and Tu-2449SC/RRV-GFP cells, respectively. Group 6, 7, and 8 received ratios of 3/97, 30/70 and 100/0 of Tu-2449SC/RRV-scFvFc-PDL1 and Tu-2449SC/RRV-GFP cells, respectively. Tumor sizes were monitored 3 times a week. Animals were removed from the study if tumors became necrotic or reached 2000 mm^3^ in volume. All mice that cleared their initial tumor implant from RRV-scFv-PDL1 or RRV-scFvFc-PDL1 treated groups (*n* = 15) and naïve group (*n* = 5) were challenged with 2 × 10^6^ Tu-2449SC on the left side of the flank. Tumor growth and measurement were monitored and recorded over time.

For the orthotopic glioma model, 8-week-old female B6C3F1 mice underwent surgical implantation of the tumor cells by Hamilton syringe. The stereotaxic coordinates were anteroposterior (AP), 0.5 mm; mediolateral (ML), 1.8 mm; and dorso-ventral (DV), 3.5 mm (from bregma). Mice in each group (*n* = 10) were intracranially (i.c.) implanted with 1 × 10^4^ of Tu-2449 cells. Survival analysis was monitored for approximately 90 days. Mice in the experimental groups were injected with purified RRV-scFv-PDL1 of 1 × 10^5^ or 1 × 10^6^ transduction unit (TU) on day 4 post tumor implant. Control groups are mice bearing 100% pre-transduced RRV-scFv-PDL1 tumor cells and Tu-2449SC tumor cells treated anti-PD-1 antibody (300 µg per mouse i.p. induction on day 4; 200 µg per mouse maintenance dose on day 10, 14 and 17) or isotype control. Survival data were plotted by the Kaplan–Meier method. Statistical significance of survival between mice treated with isotype and 100% pre-transduced scFv-PD-L1 or injection-treated RRV-scFv-PDL1 groups were determined by the Log-rank (Mantel-Cox) test using GraphPad Prism 7 (GraphPad Software, Inc.). Mice which had survived from initial tumor implant from RRV scFv-PDL1 treated groups were challenged with 2 × 10^6^ Tu-2449 cells on the right flank. Tumor growth and measurement were monitored and recorded over time.

### Analysis of tumor infiltrating lymphocytes

In a separate Tu-2449SC subcutaneous tumor model experiment, mice from each assigned group that showed measurable tumor growth were sacrificed at day 39 post tumor implantation. Tumors were excised from the skin and minced into small pieces in HBSS (ThermoFisher Scientific, # 14175095) and single-cell suspensions were obtained using gentleMACS™ C Tubes with gentleMACS™ Dissociator (Miltenyi Biotec, # 130-096-334 and # 130-093-235), and enzymatic solution containing 50 µg/mL final Liberase™ TM (Sigma-Aldrich, # 5401119001) and 200 U/mL final Deoxyribonuclease I (Sigma Aldrich, # D5025). After filtration through a 70 µm cell strainer and washing, erythrocytes were removed following incubation with eBioscience 1X RBC Lysis Buffer (ThermoFisher Scientific, # 00-4300-54). Viable cell counts were determined using a Cellometer K2 (Nexcelom Bioscience). For subsequent flow cytometry analyses, cells were first incubated with TruStain FcX antibody (BioLegend, # 101320) and Zombie UV (BioLegend, # 423108) before being subjected to surface staining with fluorochrome-conjugated antibodies against mouse CD45 (BioLegend, # 103132), CD3ɛ (BD Biosciences, # 563565), CD4 (BD Biosciences, # 564922) and CD8α (BioLegend, # 100759). Acquisition was conducted on an LSRFortessa X-20 flow cytometer and analysis by using FlowJo software (version 10) with flowAI (1.7) plugin as described [52].

### Measurement of scFv PD-L1 level in serum from tumor-bearing mice

Recombinant human PD-L1 (R&D Systems # 156-B7) was immobilized on the CM5 sensorchip surface standard amine coupling chemistry using a Biacore 3000 instrument. The running buffer comprised 25 mM HEPES, pH 7.5, 150 mM NaCl, 0.005% P_20_ (Tween 20), and 0.5 mg/mL BSA at 25° C. Purified scFv PD-L1 (>95% purity) at concentrations ranging between 0.01 200 nM (∼0.0028 µg/mL-5.6 µg/mL) was spiked into 1:20 (v/v) diluted naïve serum to generate a standard curve for every 10 regeneration cycles. Experimental samples were also prepared at 1:20 dilution and injected in at a flow rate of 5 µL/min for a 6-minute contact time. Raw data was analyzed in the Scrubber2 software (BioLogic Software). The lower limit of quantification of this assay is determined to be 0.011 µg/mL. The concentration of scFv PD-L1 level in serum was determined using the SoftMax Pro 5.45 software (Molecular Devices) with the standard curves plotted using a 4-parameter fit algorithm.

## SUPPLEMENTARY MATERIALS FIGURES AND TABLE


