# Supplementary material for: PD-L1 checkpoint blockade delivered by retroviral replicating vector confers anti-tumor efficacy in murine tumor models
Source: Oncotarget. 2019 Mar 19;10(23):2252–69. doi: 10.18632/oncotarget.26785 (PMC6481342; doi:10.18632/oncotarget.26785)
Supplement: Supplementary file 1 [file oncotarget-10-2252-s001.pdf]

PD-L1 checkpoint blockade delivered by retroviral replicating vector confers anti-tumor efficacy in murine tumor models

SUPPLEMENTARY MATERIALS

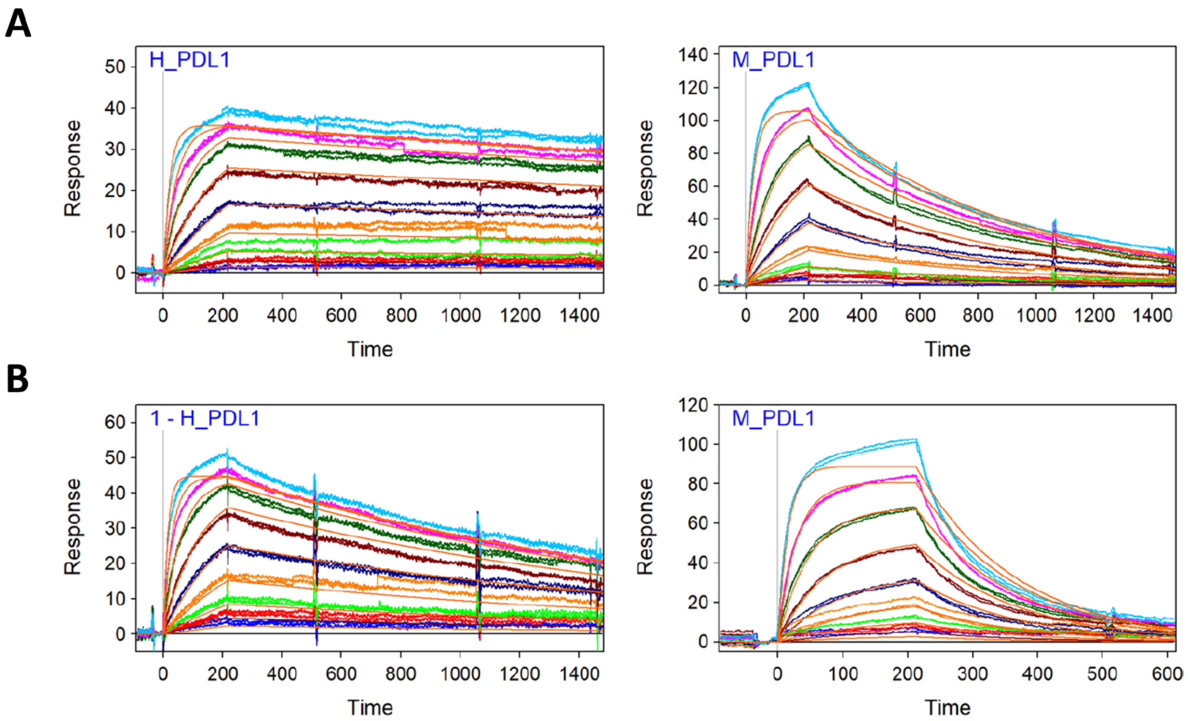

C

Antigen	Temp °C	k_{on} x 10^5 M^{-1} s^{-1}	k_{off} x 10^{-4} s^{-1}	K_D nM	$T_{1/2}$ Minutes
H_PDL1	25	**3.58 ± 0.811**	**1.51± 0.352**	**0.426± 0.065**	**77**
M_PDL1	25	**2.93 ± 0.343**	**13.9 ± 0.45**	**4.78 ± 0.065**	**8.3**
H_PDL1	37	**5.28**	**6.27**	**1.21**	**18.4**
M_PDL1	37	**5.18**	**65.3**	**12.6**	**1.8**

Supplementary Figure 1: Binding affinity of purified scFv PD-L1 protein against recombinant human and mouse PD-L1. Binding affinities were measured at (**A**) 25° C or (**B**) 37° C by surface plasmon resonance using a GE BIAcore 3000 instrument. Recombinant human PD-L1-Fc (R&D Systems, #156-B7) and recombinant mouse PD-L1-Fc (R&D Systems, # 1019-B7) were coated on CM5 biosensor chips. For kinetics measurements, a 2-fold serial dilutions were injected in SPR running buffer (150 NaCl, 25 HEPES 7.5, 10% glycerol 0.005% P20 1% DMSO) at ambient temperature with a flow rate of 50 µL/min. Association rates (ka) and dissociation rate (kd) were calculated using a simple one-to-one Languir binding model (BIAcore Scrubber 2 software). The equilibrium dissociation constant (kD) was calculated as the ratio of kd/ka. (**C**) Summary of kinetic parameters for scFv PD-L1 binding human and mouse PD-L1 at 25° C and 37° C. Mean values and standard deviation indicated for the data sets collected at 25° C were from 3–6 independent experiments.

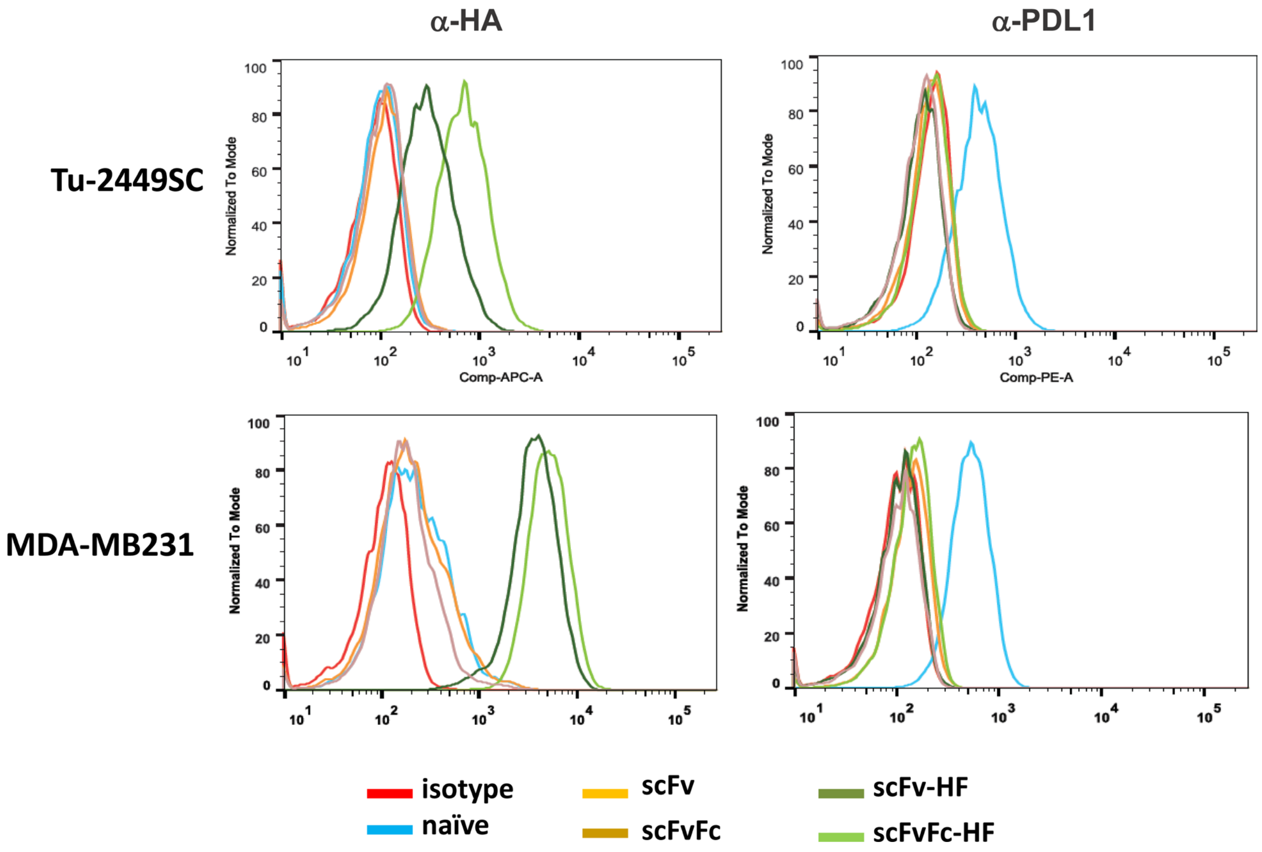

Supplementary Figure 2: Binding specificity of scFv-PD-L1 to PD-L1. scFv PD-L1 bystander *trans*-binding activity to PD-L1 on the cell surface. IFNγ-treated Tu-2449SC and MDA-MB231 cell lines maximally infected with RRV scFv-HF PDL1 (HA-tagged scFv PD L1) were stained with Alexa Fluor 647-conjugated anti-HA antibody and PE-conjugated anti-human PD L1 antibody. HA-positive, PD L1-positive cells were properly compensated and gated for flow cytometric analysis. Naïve indicates background signal obtained from unstained naïve cells. Isotype signal indicates signal obtained from naïve cells stained with isotype antibody.

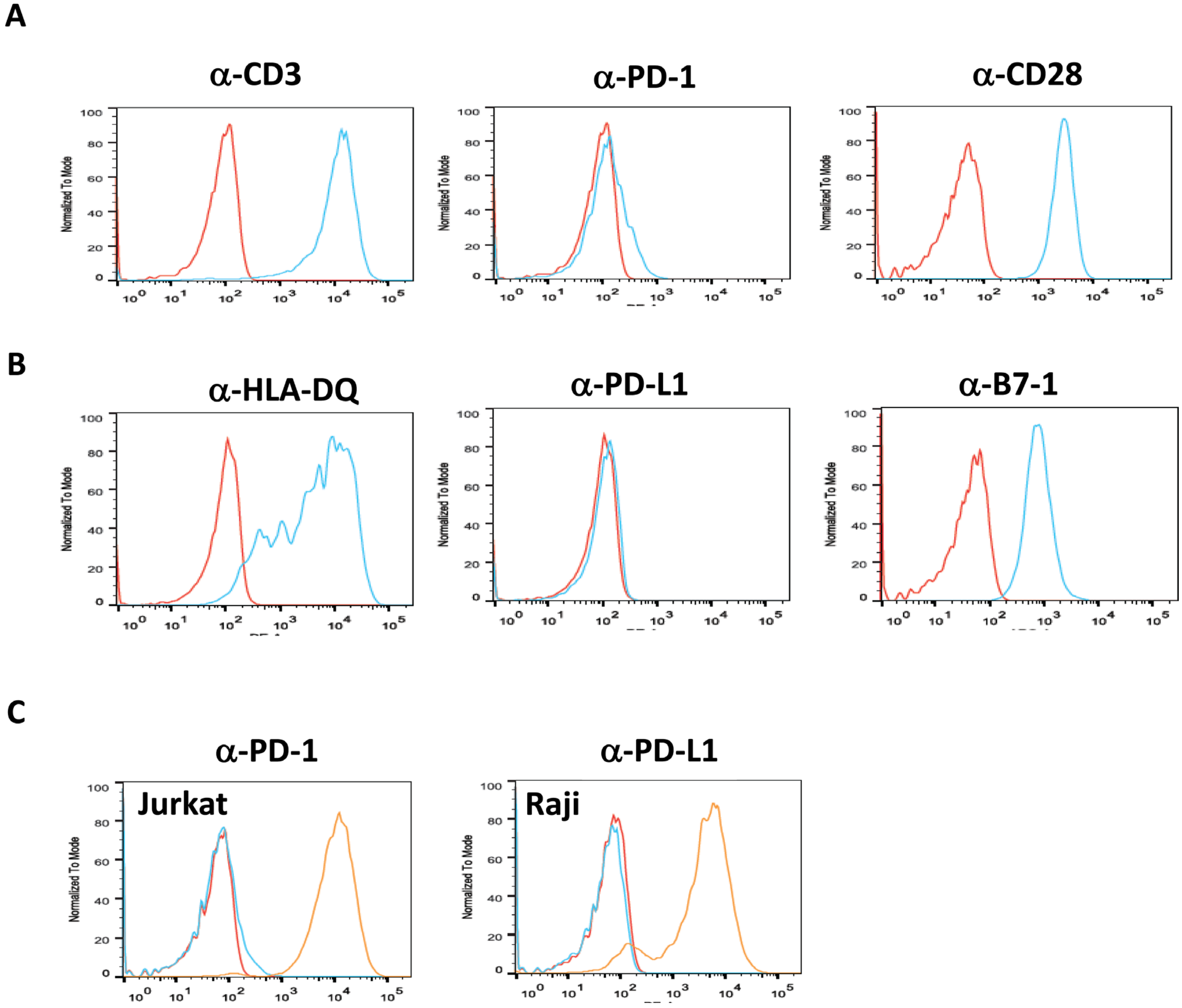

Supplementary Figure 3: Cell surface expression profile of parental Jurkat T cells and Raji B cells. Histograms showing the expression of (**A**) CD3 (BioLegend, #300308), PD-1 (BioLegend, #329908) and CD28 (BioLegend, #302912) in parental Jurkat T cells and (**B**) HLA-DQ (BioLegend, #318106), PD-L1 (eBioscience, #12-5983) and B7-1 (BioLegend, #305220) in Raji B cells. Red-lined histograms are isotype controls. (**C**) PD-1 expressing Jurkat and PD-L1 expressing Raji B cells after transduction with lentiviral vector encoding PD-1 and PD-L1, respectively. Red-lined histograms are isotype controls; blue-lined histograms are antigen staining of parental cells and orange-lined histograms are PD-1-overexpressing Jurkat T cells or PD-L1-overexpressing Raji B cells.

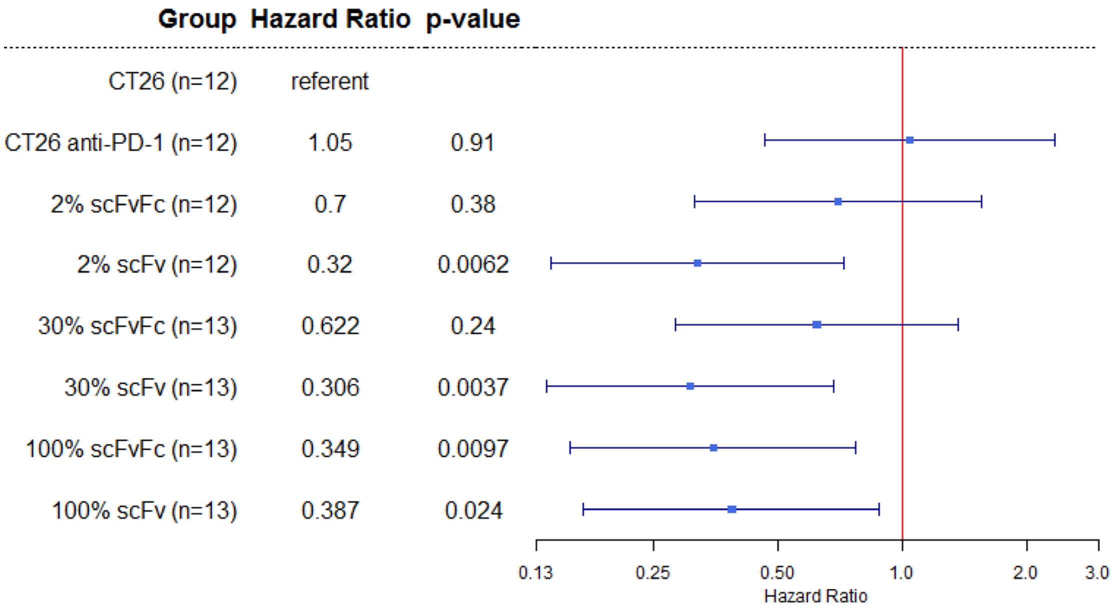

Supplementary Figure 4: Subcutaneous syngeneic tumor model with pre-transduced CT26 cells expressing scFv PD-L1 or scFvFc PD-L1 demonstrates anti-tumor activity. BALB/c mice were implanted subcutaneously with 5E5 of either 100% CT26-WT, 2% CT26 pre-transduced + 98% CT26 WT, 30% CT26 pre-transduced + 70% CT26 WT, or 100% pre-transduced CT26 tumor cells on the right flank in 100 µL volume. For mice received αPD-1 isotype antibody (Armenian hamster polyclonal IgG) and αPD-1 antibody (Clone J43), antibodies were administered when the tumor reached 50–100 mm^3 with 300 µg/mouse induction dose for their first treatment and 200 µg/mouse as a maintenance dose for the 6 subsequent treatments every 3 days. Tumors were monitored 2–3 times a week until tumor volumes reach ~2000 mm^3 at which animals were removed from study. Forest plot showing the results of a Cox proportional hazards regression model investigating the association between survival time of tumor-bearing mice with eight different treatment groups (n = 12–13 mice per group). Hazard ratios for each treatment condition relative to untreated CT26 mice are displayed in the table to the left, and are plotted on the right as blue squares with blue lines showing 95% confidence intervals. HRs less than one indicate improved outcomes for the designated group relative to the untreated CT26 group (referent). A red vertical line indicates the location of a hazard ratio equal to 1.0, and significance is achieved if the 95% CI does not cross this line. Log-rank p-values are displayed. Analysis was performed with R statistical software (version 3.4.1) using the survival, epitools and forest plot packages.

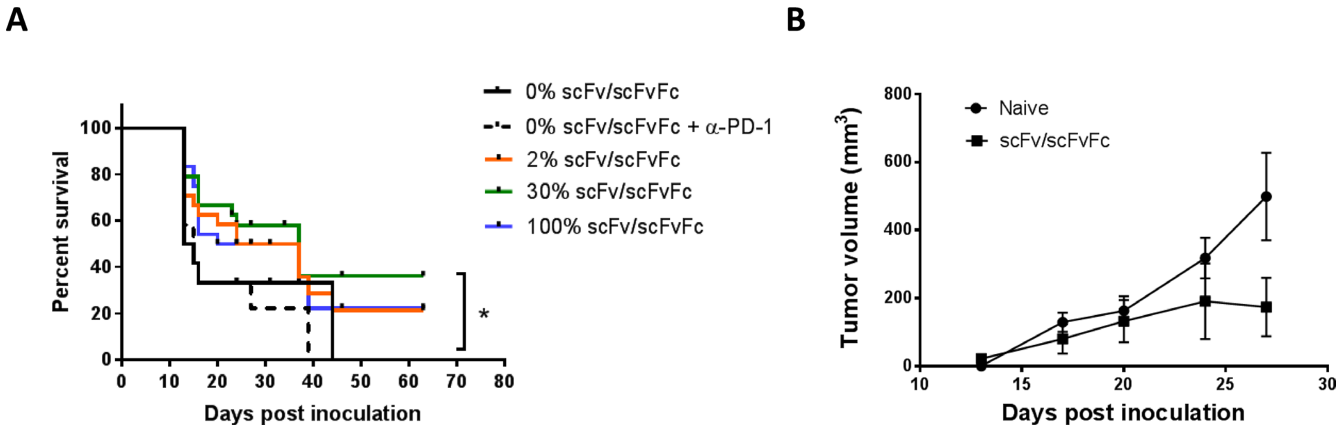

Supplementary Figure 5: Survival benefit and anti-tumor response in an orthotopic breast cancer model. (**A**) Mixture of 5×10^5 EMT6 tumor cells pre-transduced with RRV-scFv-PDL1, RRV-scFvFc-PDL1 or RRV-GFP at indicated ratios were implanted in the mammary fat pad in 8 week old BALB/C female mice (n = 10 per group). Mice with 0% scFv/scFvFc carry tumors pre-transduced with 100% RRV-GFP. Survival was monitored for 90 days and survival data were plotted by the Kaplan–Meier method. Anti-PD-1 antibody was included as a control and was i.p. administered on day 10 (300 µg per mouse), day 13, day 16, and day 19 (200 µg per mouse). Statistical significance of survival between mice treated with anti PD-1 antibody and RRV scFv PDL1 treated mice was determined by the Log-rank (Mantel-Cox) test, $^*p = 0.0271$. Ticks on the graph indicate mice censored due to tumor necrotic and were terminated; the graph does not exclude these mice. (**B**) Naïve mice (n = 5) and mice that survived initial tumor implant from RRV scFv PDL1 or RRV scFvFc PDL1 treated groups (n = 5) were challenged with 1×10^6 EMT6 tumor cells on the flank and tumor growth was monitored overtime. Error bars indicate SEM of the dataset.

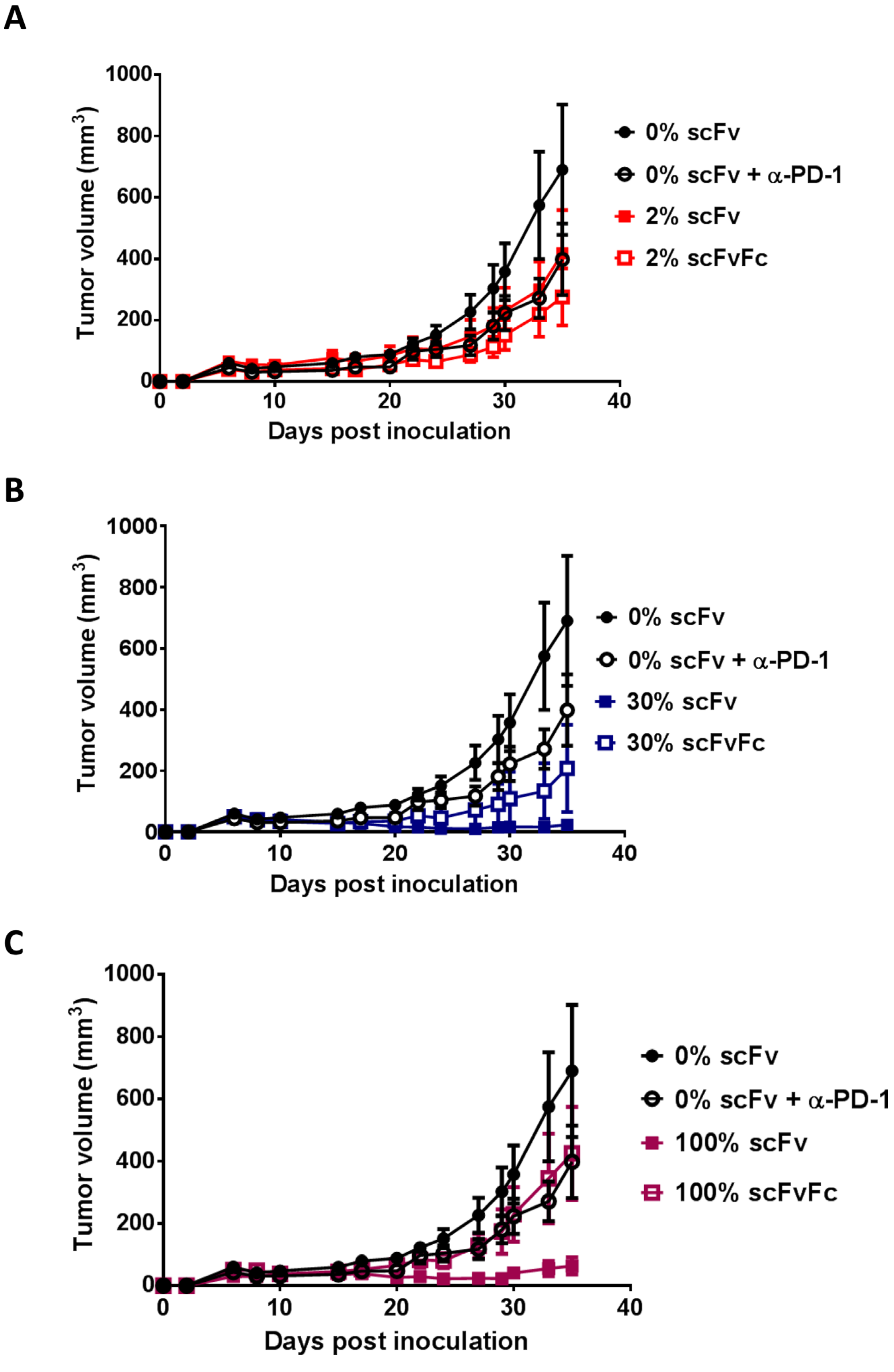

Supplementary Figure 6: Subcutaneous tumor model with pre-transduced Tu 2449SC cells expressing scFv PD-L1 demonstrates a dose-dependent anti-tumor activity. (**A**) Tu 2449SC cells maximally infected with RRV scFv-PDL1 or RRV-GFP at indicated ratios were subcutaneously implanted on the right flank of 8 week old female B6C3F1 mice and assigned to indicated groups (*n* = 10 per group). Control groups are mice implanted with 100% Tu 2449SC/RRV GFP cells received PBS or anti-PD-1 antibody. Anti-PD-1 antibody (Clone J43) was i. p. administered on day 0 (300 µg per mouse), day 3, day 6 and day 9 (200 µg per mouse). Tumor sizes were monitored 3 times a week until tumor volumes reach sizes >1000 mm^3.

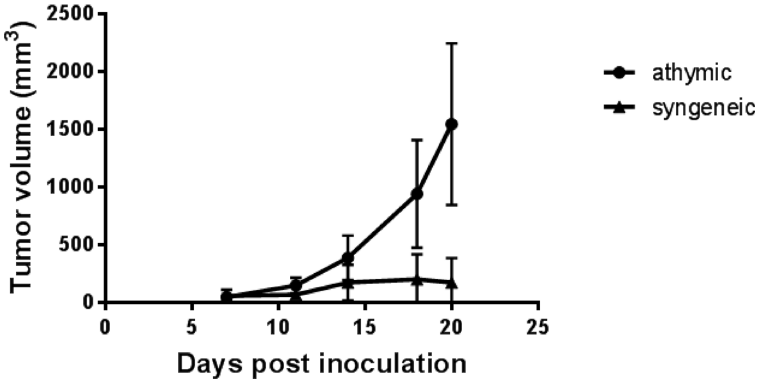

Supplementary Figure 7: Anti-tumor effect of scFv PD-L1 expressed from RRV-scFv-PDL1 infected tumor is immune mediated. Spider plot of tumor growth in B6C3F1 syngeneic and athymic mice (Envigo), two-million Tu-2449SC tumor cells 100% pre-transduced with RRV-scFv-PDL1 were implanted subcutaneously in B6C3F1 mice (n = 10 per group) and athymic mice (n = 10). Tumor growth was monitored over time. Error bars indicate the standard error (SEM) of the dataset.

A

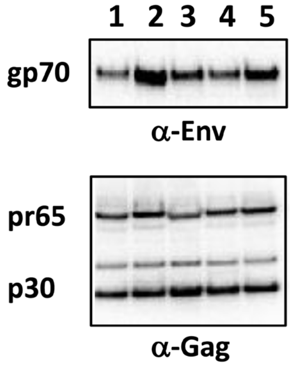

B

	TU/mL	Std Dev
RRV-scFv-PDL1	5.10E+06	1.15E+06
RRV-scFv-HF-PDL1	7.53E+06	1.69E+06
RRV-scFvFc-HF-PDL1	4.68E+06	8.66E+05
RRV-scFv-HF-PDL1	8.05E+06	2.19E+06
RRV-IRES-GFP	5.81E+06	7.57E+05

C

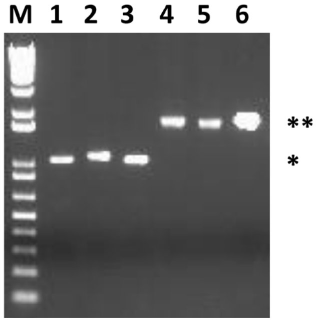

Supplementary Figure 8: Viral envelope protein expressed from RRV-scFv-PDL1 is properly processed and produces high titers in Tu-2449 cells. (**A**) Western blot analysis of viral proteins produced from maximally infected Tu-2449 cells. One milliliter of 0.45 µm filtered viral supernatants was centrifuged through a 20% sucrose cushion at 14,000 rpm for 1 h. The viral pellets were resuspended in 20 µL XT loading buffer and resolved in a 4–12% gel. Membranes were incubated with anti-MLV Env antibody 83A25 which detects the gp70 domain of the viral envelope protein (top panel); anti-gag antibody which detects unprocessed (Pr65) and processed gag polyprotein (p30) (bottom panels) as included as a loading control. Lane 1: RRV-scFv-PDL1; lane 2: RRV-scFv-HF-PDL1; lane 3: RRV-scFvFc-HF-PDL1; lane 4: RRV-scFvFc-PDL1; lane 5: RRV-GFP which was included as a positive control. (**B**) Same viral supernatants were used to perform titer assay as described (Lin et al., 2017). RRV-GFP was included as a positive control. (**C**) Stability of 2A-scFv-PDL1 transgene cassettes from proviral DNA in maximally infected Tu-2449 cells (14 days post infection). End-point PCR with primer set spanning the transgene cassette was performed as described (Lin et al., 2017). *indicates size of the PCR product expected for the intact 2A-scFv-PDL1 and 2A-scFv-HF-PDL1 transgene cassette (lane 1 and 2); positive control (lane 3) is pAC3-scFv-PDL1 plasmid DNA. **indicates size of the PCR product expected for the intact 2A-scFvFc-PDL1 and 2A-scFvFc-HF-PDL1 transgene cassette (lane 4 and 5); positive control (lane 6) is pAC3-scFvFc-PDL1 plasmid DNA. M indicates the DNA molecular ladder (1 Kb Plus, Life Technologies).

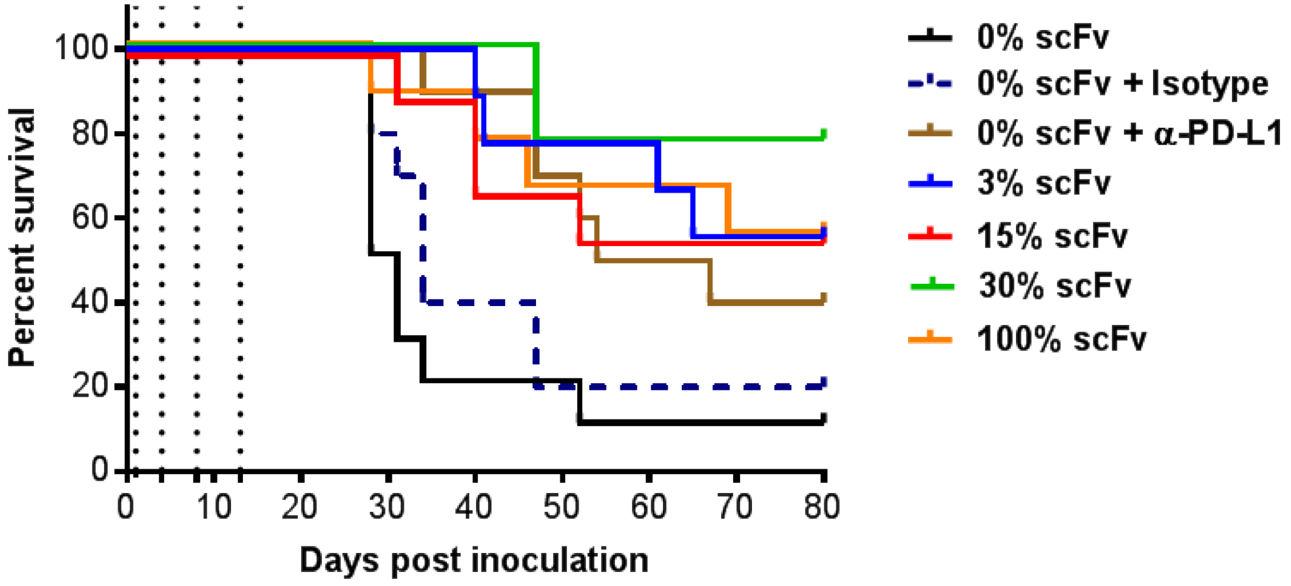

Supplementary Figure 9: Kaplan–Meier survival analysis of a syngeneic orthotopic glioma model. Pre-transduced GL-261 tumor cells with RRV-scFv-PDL1 or RRV-GFP were mixed at various ratios (RRV-scFv-PDL1/RRV-GFP at 3/97, 15/85, 30/79, 100/0) and implanted intracranially in C57BL/6 mice (*n* = 10 per group). Mice implanted with GL-261 tumor cells pre-transduced with RRV-GFP and treated with anti-PD-L1 antibody (Clone 10F.9G2, indicated as 0% scFv + aPD-L1) were included as a control in the experiment. Dashed vertical lines indicate anti-PDL-1 antibody (Clone 10F.9G2; 200 µg/mouse) i. p. administered on day 1, 4, 8, and 13.

Supplementary Table 1: Titers of various RRV-scFv-PDL1 vectors produced from transiently transfected 293T cells

	TU/mL	Std Dev
RRV-scFv	2.09E+06	4.80E+05
RRV-scFv-HF	2.08E+06	6.73E+05
RRV-scFvFc	1.98E+06	4.38E+05
RRV-scFvFc-HF	1.29E+06	1.87E+05
